# OFF-responses of interneurons optimize avoidance behaviors depending on stimulus strength via electrical synapses

**DOI:** 10.1371/journal.pgen.1007477

**Published:** 2018-06-25

**Authors:** Sayaka Hori, Shigekazu Oda, Yuji Suehiro, Yuichi Iino, Shohei Mitani

**Affiliations:** 1 Department of Physiology, Tokyo Women’s Medical University School of Medicine, Tokyo, Japan; 2 Department of Biological Sciences, Graduate School of Science, The University of Tokyo, Tokyo, Japan; HHMI, UC San Diego, UNITED STATES

## Abstract

Optimization of the types and timing of avoidance behaviors depending on the intensity of a noxious stimulus is essential for survival; however, processing in the central nervous system and its developmental basis are largely unknown. Here, we report that *Caenorhabditis elegans* preferentially selects one of three different types of avoidance behaviors depending on the strength of the noxious stimulus. We screened 210 neuronal transcription factors using a combination of optogenetics and RNA interference methods and identified 19 candidates required for avoidance behaviors. One candidate, gene *lin-32 (abnormal cell* *LIN**eage* *32**)*, which encodes an *atonal* homolog, is required for the neural fate determination of AIB interneurons and functions by regulating the expression of electrical and chemical synapse genes, namely, *inx-1* (*innexin 1*) and AMPA-type ionotropic glutamate receptor *glr-1*. When examined by Ca imaging, AIB showed an OFF calcium increase to the noxious stimulus. The OFF calcium increase was provoked only by strong stimulation, suggesting a role for optimization of the avoidance behavior. However, *lin-32* mutants showed an impaired AIB OFF calcium increase, concomitant with a reduced occurrence of the dynamic avoidance behavior called the "omega turn". The AIB neural responses may be transferred to downstream inter/motor neurons projecting to the neck muscles via electrical synapses comprising *inx-1*. Finally, we found a correlation between powerful contractions of the neck muscles and omega turns. Thus, the central regulation of the magnitude and timing of activation of the AIB interneurons optimizes the probability of omega turn depending on the stimulus context.

## Author summary

For survival, animals exhibit appropriate behaviors depending on the type and strength of a stimulus; however, processing in the central nervous system and its developmental basis are largely unknown. Here, we performed RNA interference screening of 210 transcription factors by observing the avoidance behaviors evoked by a harmful stimulus and identified 19 candidates with behavioral defects. One candidate gene, *lin-32*, which encodes a homologue of a well-conserved transcription factor, *atonal*, that plays a key role in cell type specification, is required for the optimization of avoidance behaviors. *lin-32* regulates the expression of various synaptic genes in the key neurons required for the optimization of avoidance behaviors. The key neurons respond at the time of the removal of the strong stimulation, and an appropriate magnitude and timing of the calcium increase are required to determine the type and timing of dynamic avoidance behaviors. However, the key neuronal response does not occur in the *lin-32* mutants. Activation of the key neurons may be transferred to the downstream neurons projecting to the neck muscles via electrical synapses. Finally, we show a correlation between powerful neck muscle contractions and dynamic avoidance behaviors.

## Introduction

Animals optimize a behavioral response, such as the mode, strength or duration of the stimulus at the proper time, to sensory stimuli depending on context [1]. Specifically, the optimization of avoidance behaviors from harmful stimuli is a conserved function that is essential for the survival of various animal species ranging from invertebrates to humans. Although simple all-or-none reflex responses are well documented for many behaviors, including avoidance behaviors such as touch responses [2,3,4,5,6], the understanding of optimization of avoidance behaviors becomes complicated when apparently similar noxious stimuli induce distinct behavioral patterns. Such behaviors may likely involve the central nervous system to develop diverse behavioral repertoires [7,8], and defects in avoidance circuit formation and/or function in the central nervous systems may lead to a life crisis or a bias of the sensory behavior [9]. Thus, an elucidation of the neural and molecular basis is an important challenge.

**Fig 1:**
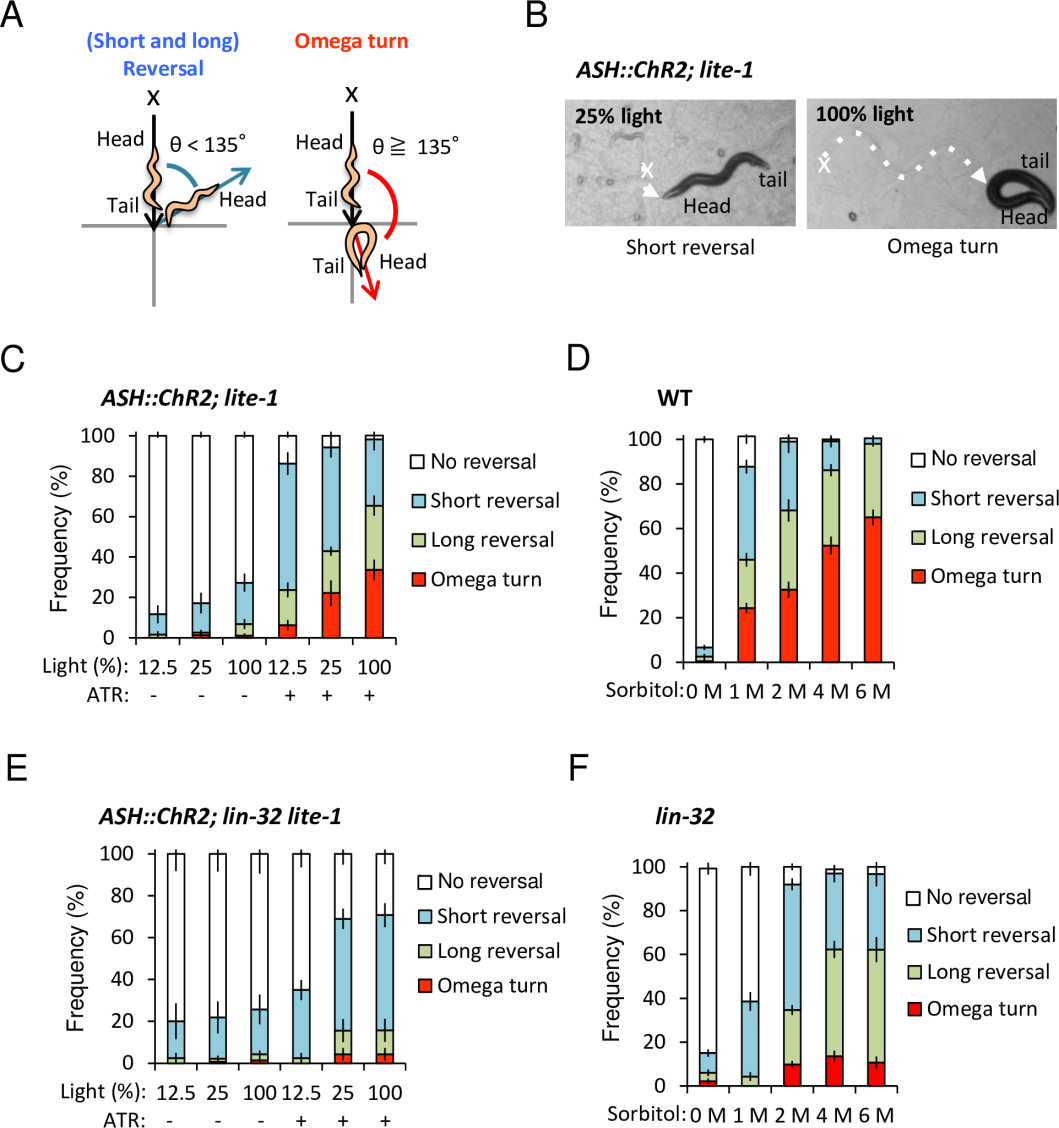
*lin-32* is required for proper optimization of probability of omega turn with proportional stimulus strength. (A) A scheme of the avoidance behaviors [11]. After contacting a noxious stimulus (x), an animal exhibits backward behavior. Short and long reversal are subdivided by the backward length with 1~2 head swings or three head swings or greater without omega turn, respectively. An omega turn is specifically defined by reorientation angles (θ ≧135) following the backward behaviors with/without either short reversal or long reversal. (B) *lite-1* mutants expressing ChR2(H134R) in ASH sensory neurons (ASH::ChR2(H134R); *lite-1*) exhibit short reversal (left panel, dashed line) with 25% light irradiation (x) and omega turns (right panel, dashed line) with 100% light irradiation (x). (C) *ASH*::*ChR2(H134R); lite-1* animals with ATR can optimize their dominant behaviors from reversals to omega turns with proportional to light intensity. n = 6,7,9,8,7,11. (D) Wild type animals adjust probability of omega turns depending on the sorbitol drop concentration. n = 17,30,20,20,20. (E) ASH::ChR2(H134R); *lin-32 lite-1* animals showed reduced omega turns even with ATR and 100% light intensity. n = 4,7,7,4,7,7. (F) *lin-32* animals show reduced omega turns independent on the sorbitol drop concentration. n = 20,7,33,20,12. n = plate (cohort) of approximately 10–20 animals. The data are presented as the mean ± SEM.

A simple model animal, *C*. *elegans*, basically exhibits three types of avoidance behaviors, namely, the short reversal, long reversal, and omega turn, upon direct contact of the head region with a noxious stimulus [10]. In all cases, backward behaviors occur upon the initiation of all types of avoidance behavior (Fig 1A). Short and long reversals are classified according to the backward length. Both reversals briefly interrupt forward movement but the animal still maintains its original heading. On the other hand, the omega turn is classified according to the large reorientation angle (>135°) accompanied by any backward length, which the animal reorients towards the original position [10]. *C*. *elegans* must weigh the physical risks due to the nociceptive stimulus and it should choose the deliberate way of avoidance behaviors.

The basic avoidance circuit of *C*. *elegans* has been clarified [11]. ASH nociceptive neurons mainly receive noxious stimuli, for example, high osmolarity, harsh touch, heavy metals, and dense odorants, detergents etc. [4], and regulate avoidance behaviors through two downstream circuits, namely, the primary circuit and the secondary circuit [11]. In the primary circuit, ASH forms chemical synapses with AVA interneurons [12]. AVA contributes to the frequency and length of backward movement through an excitatory connection to DA and VA motor neurons; in this way, AVA may determine the short and long reversals [13,14,15,16]. In development, transcription factor *fax-1 (defective* *FA**sciculation of a**X**ons 1)*, which is an ortholog to the vertebrate *PNR* (*P**hotoreceptor-specific* *N**uclear* *R**eceptor*), regulates the differentiation of AVA [17].

In the secondary circuit, ASH directly forms chemical synapses with AIB interneurons [12]. AIB determines the length of backward movement and frequency of omega turns; in this way, ASH determines all three types of avoidance behaviors (the short reversal, long reversal, and omega turn) [15]. AIB activity can affect variability in the AVA neural response via chemical synapses as an interference circuit [18]. The primary and secondary circuits should eventually coordinate muscle contractions via motor neurons [12]. Since the probabilities of avoidance behaviors vary depending on context, the integration process through the neural circuit described above is unknown and needs further study.

In this study, we report the neural and molecular mechanisms underlying the optimization of avoidance behaviors depending on the intensity of a mono-modal noxious stimulus in *C*. *elegans*. We found that the probabilities of each response depended on the intensities of noxious stimuli: weak stimuli often resulted in short or long reversals, and strong stimuli often resulted in an omega turn. Then, we performed RNAi screening 210 neural transcription factors and identified 19 candidates that impaired avoidance behavior. One of them, proneural gene *atonal* homolog, *lin-32*, regulates the formation or function of electrical and chemical synapses in AIB interneurons, which are key neurons for the optimization of probability of omega turn via the regulation of neck muscle contraction presumably via electrical synapses. We clarified a series of integration processes by the central nervous system to induce distinct avoidance patterns in response to mono-modal sensory stimuli depending on the context at the appropriate time.

## Results

### *C*. *elegans* optimizes the probabilities of avoidance behaviors depending on the stimulus strength

To clarify how *C*. *elegans* optimizes avoidance behaviors by selecting one of three types of behaviors depending on the strength of a mono-modal noxious stimulus (Fig 1A), we used optogenetics to activate only the ASH-mediated neural circuit and apply quantitatively controlled stimulation (12.5%, 25%, and 100% blue light) [15]. The results showed that the probability of omega turns increased with the increasing intensities of stimulation in the presence of OP50 containing all-trans-Retinal (ATR) and peaked under the strongest stimulus (100% light) with ATR (Fig 1B and 1C, S1 Movie). The similar result was obtained in an experiment using no-food plates (S1A Appendix). In both cases, there are significant differences between 0% or 12.5% and 100% light with ATR (p < 0.05, ANOVA followed by the Tukey's post hoc tests). Most of the control animals without ATR or without ChR2 (*lite-1* mutants with no transgene) showed no avoidance behavior (Fig 1C, S1A and S1B Appendix).

Next, to determine whether the same effect was observed with natural ASH-activating stimuli, we performed the drop test using different concentrations (0, 1, 2, 4, and 6 M sorbitol, or 0, 1, 2, and 4 M glycerol) of high-osmolarity solutions as noxious repellents. Again, the frequency of omega turns significantly increased proportional to the stimulus intensity (Fig 1D, S1C Appendix). In both cases, there are significant differences compared 1 M vs. 4 M and/or 6 M, 2 M vs. 4 M and/or 6 M solutions) (p < 0.01, ANOVA followed by the Tukey's post hoc tests). Pokala et al had showed shifts in the backward length distribution as a function of glycerol concentration [16]. Our results showed that the strong stimulus tends to induce omega turns, providing additional insight into the optimization of probability of omega turn depending on the strength of a single type of stimulus in *C*. *elegans*.

Sorbitol solution is less viscous in higher concentrations than glycerol solution, it is easier to examine this type of behavior. Accordingly, we chose to use sorbitol for subsequent experiments.

### *atonal* homolog *lin-32* regulates the differentiation of AIB interneurons, which are required for the optimization of probability of omega turn

To understand the molecular and neural basis for optimization of the avoidance circuit in *C*. *elegans*, we screened 210 neuronal transcription factors with a combination of optogenetics and enhanced neuronal RNAi methods [19] (S1 Table). The results showed that RNAi knockdown of 19 candidate genes was associated with reduced avoidance behavior (S2 Table). Next, to confirm the RNAi phenotype, we tested a mutant of each candidate gene by optogenetics and high-osmolarity ring assay, and the top ten common genes, namely, *lin-32*, *unc-130*, *ham-2*, *lin-11*, *fax-1*, *unc-86*, *ceh-17*, *xnd-1*, *fozi-1*, and *lsy-2*, were predicted to affect the formation and/or functions of the avoidance circuit.

In this study, we have focused on one candidate gene, *lin-32*, because *lin-32* is a homolog of *Drosophila atonal* and mammalian *atoh1/ato1/Math1*, which is one of the most important and well-conserved proneural genes for neurogenesis [20]. We used optogenetics to analyze whether *lin-32* null mutants selectively expressing ChR2(H134R) in ASH sensory neurons could optimize avoidance behaviors. As a result, *lin-32* mutants exhibited impaired omega turns with and without food (Fig 1E, S1D Appendix, S2 Movie), showing no significant increase depending on the stimulus strength (p > 0.05, ANOVA followed by the Tukey's post hoc tests). Compared with those of the wild type animals, there was significant decrease in the frequency of the omega turns at 100% light (p < 0.0001, ANOVA followed by the Tukey's post hoc tests). This phenotype was also observed with the drop test using sorbitol and glycerol solution (Fig 1F, S1E Appendix), suggesting the involvement of *lin-32* in the optimization of a probability of omega turn corresponding to general high-osmolarity.

### *lin-32* is required for the proper function of AIB interneurons

We first estimated simple ASH differentiation defect in *lin-32* mutants, but ASH morphology and differentiation appear normal using multiple differentiation markers (S2A, S2B, S2D, S2E, S2G and S2H Appendix, S3 Table) (p > 0.05, Fisher's exact test). Dye-filling analysis suggests functional intraflagellar transport in ASH (S2D and S2E Appendix, S3 Table) (p > 0.05, Fisher's exact test). Normal expression of *eat-4* marker supports normal glutamatergic synaptic transmission of ASH (S2G and S2H Appendix, S3 Table) (p > 0.05, Fisher's exact test).

**Fig 2:**
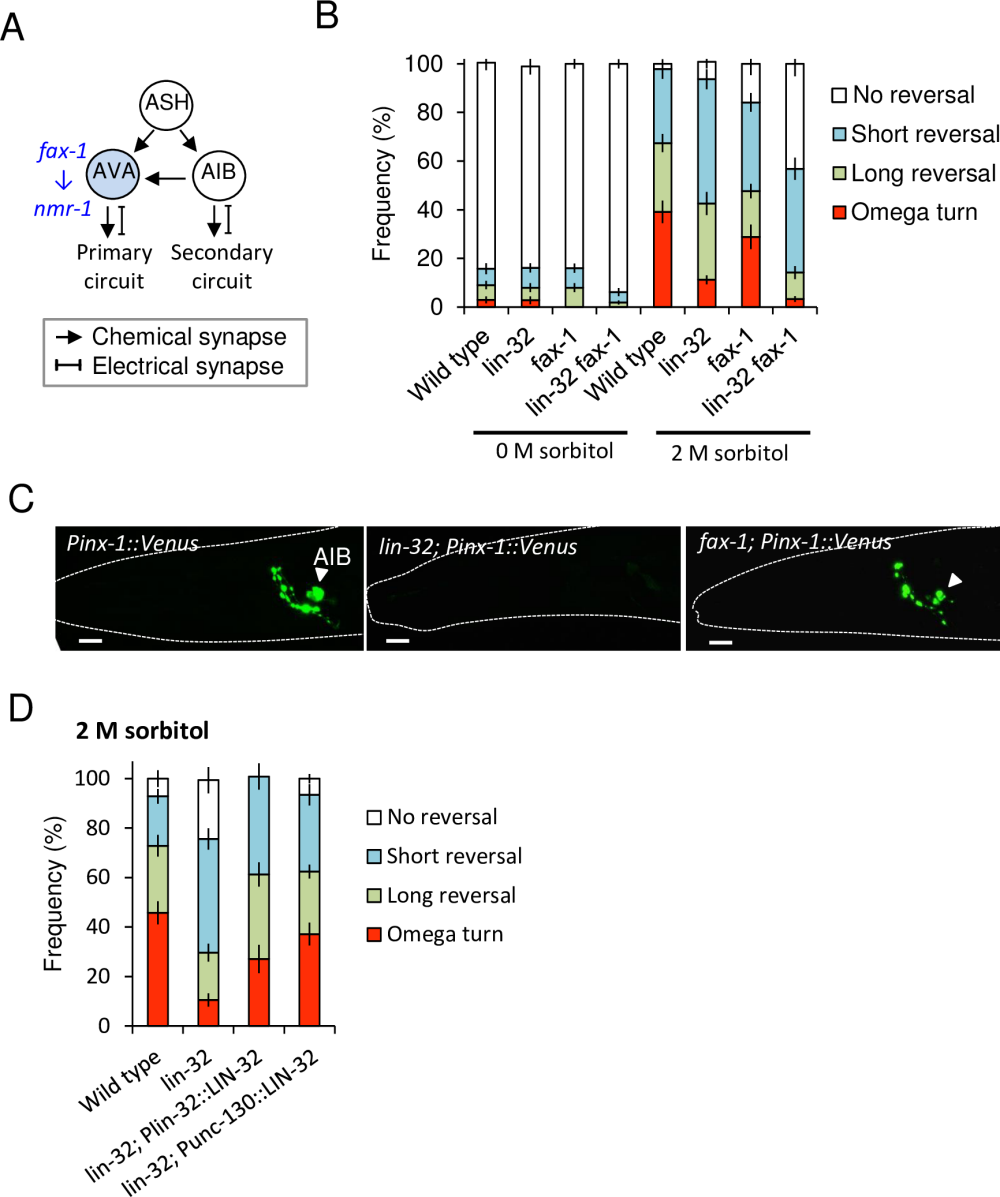
*lin-32* is required for the determination of AIB interneurons, which are the key neurons for the optimization of probability of omega turn. (A) A scheme of the first central layer interneurons in the primary and secondary circuits. ASH directly connects to AVA and AIB via chemical synapses. The *fax-1* gene induces expression of *nmr-1* in AVA. (B) *fax-1* mutants show a partial avoidance defect to 2 M sorbitol, and *lin-32 fax-1* double mutants show more reductions in omega turns and long reversals than the *lin-32* and *fax-1* single mutants. n = 20,19,20,21,20,17,15,17. (C) Wild type animal selectively expresses an electrical synapse gene, *inx-1*, in its AIB neurons (arrowhead), but a typical *lin-32* mutant fails. *fax-1* mutant normally expresses *inx-1* (arrowhead). (D) The extrachromosomal expression of *lin-32* cDNA driven by both its own promoter *Plin-32* and AIB lineage-selective promoter *Punc-130* fully rescues avoidance behaviors in response to 2 M sorbitol. n = 21,21,13,16. n = plate (cohort) of approximately 10–20 animals. The data are presented as the mean ± SEM.

ASH sensory neurons evoke avoidance behaviors through the downstream primary circuit and the secondary circuit via the first layer interneurons AVA and AIB, respectively (Fig 2A). Considering the partial avoidance defect in *lin-32* mutants (Fig 1E and 1F, S1D and S1E Appendix), we hypothesized that *lin-32* may exhibit defect(s) in only one circuit. To test this hypothesis, we focused on a transcription factor, namely, *fax-1*. A previous study showed that *fax-1* is required for the expression of *nmr-1* in AVA (and AVD) interneurons in the avoidance circuit, but there is no evidence that the *fax-1* defect is restricted to AVA/D in avoidance circuit [17]. In our RNAi screening, down regulation of *fax-1* also showed partial avoidance defects (S1 and S2 Tables). First, we analyzed whether ASH differentiation of *fax-1* mutants is normal or not. As a result, *fax-1* mutant showed normal ASH morphology and expression of a marker and dye-filling (S2C and S2F Appendix, S3 Table) (p > 0.05, Fisher's exact test). Then we expected that partial AVA differentiation defects in *fax-1* mutants might cause the partial avoidance defect, and double mutant of *lin-32* and *fax-1* might show a severe defect when *lin-32* works in the parallel circuit with *fax-1*. As a result, frequency of omega turn of the double mutants was significantly impaired than *fax-1* single mutants in 2 M sorbitol condition (Fig 2B) (p < 0.0001, ANOVA followed by the Tukey's post hoc tests), we then predicted that *lin-32* might act in the secondary circuit.

Then, we examined the differentiation of AIB interneurons, which are the central interneurons of the secondary circuit. First, we observed the expression of an AIB marker, *inx-1* promoter-driven *Venus (Pinx-1*::*Venus)*. *inx-1* is a gap junction gene that is selectively expressed in AIB interneurons in the adult stage to form electrical synapses [21], but only 23.3% of *lin-32* mutants expressed *inx-1* (Fig 2C, S3 Table). Another AIB marker *Pglr-1*::*gfp* [22], which is an AMPA-type ionotropic glutamate-receptor-promoter-driven GFP, was expressed only in 4.2% of the *lin-32* mutants (S3 Table). There were significant differences compared between wild type animals or *fax-1* mutants and *lin-32* mutants using both markers (Fig 2C, S3 Table) (p < 0.05, Fisher's exact test).

In addition, we analyzed AIA and RIM interneurons in the secondary circuit; and AVA, AVD, and AVE interneurons and DA/VA motor neurons in the primary circuit using differentiation markers, but all appeared normal in *lin-32* mutants and *fax-1* mutants except for *nmr-1* expression in AVA as previously reported (S2I–S2Q Appendix, S3 Table) [17]. There are no significant differences compared with wild type animals (p > 0.05, Fisher’s exact test). These results suggest that *lin-32* is required for at least differentiation of AIB interneurons in avoidance circuits.

To support this hypothesis, we performed AIB-lineage rescue of *lin-32* using Promoter *Punc-130* (*UNC**oordinated* *130*), which has relatively AIB-restricted expression in ABp(l/r)aapap lineage including AIB precursor cells and ABp(l/r)papp(p/a) lineage including ASH precursor [23]. As a result, full-length *lin-32* cDNAs driven by the promoter *Punc-130* completely rescued the behavioral defects in the *lin-32* mutants to the same level as that of the *lin-32*-promoter-rescued animals. There is no significant difference between both rescue lines and wild type animals in terms of penetrance (p > 0.05, ANOVA followed by the Tukey's post hoc tests), and there is a significant difference between both rescue lines and *lin-32* mutants (p < 0.05, ANOVA followed by the Tukey's post hoc tests) (Fig 2D).

Lack of transcription factors sometimes results in a conversion to other cell fate in the sister lineage [24]. Indeed, in the *lin-32* mutants, the ectoblasts of the V5 lineage transforms into the V4 lineage [25]. To examine whether the AIB marker-negative cells were transformed into other cell types in the sister lineage of *lin-32* mutants (S3A Appendix), we observed the cell numbers and morphology of ASI, AWA and ASG sensory neurons using differentiation markers of each cell type. The morphology and the cell numbers were unchanged (S3B–S3H Appendix), suggesting that AIB marker-negative cells were not transformed into other neurons in the lineage. We could not determine whether the AIB cells disappeared or differentiated into cells of uncertain types. AIB marker-positive cells, which were observed in 4–23% of the *lin-32* mutants (S3 Table), were likely committed to the AIB cell fate but not sister cells.

On the other hand, AIB marker-positive cells in the *lin-32* animals suffered from functional reduction because the *lin-32* animals with such cells showed identical avoidance defects in high osmolarity ring assay to those of the animals that did not express the AIB marker (S4 Appendix). In the ring assay, wild type animals go out of the water ring, so the score of "Nematodes inside the ring (%)" is low (40%) (S4 Appendix). On the other hand, they avoid 2 M sorbitol and they cannot go out of the 2 M sorbitol ring, so the score is close to 100% (S4 Appendix). *osm-6* (*OSM**otic avoidance abnormal* *6*) mutants go out of the ring of 2 M sorbitol because the mutants cannot sense hyperosmolarity (S4 Appendix). Both *lin-32* mutants with and without expression of AIB marker show a partial defect like the result of a drop test (S4 Appendix).

### OFF calcium increase of AIB interneurons correlates with strong stimulus strength

**Fig 3:**
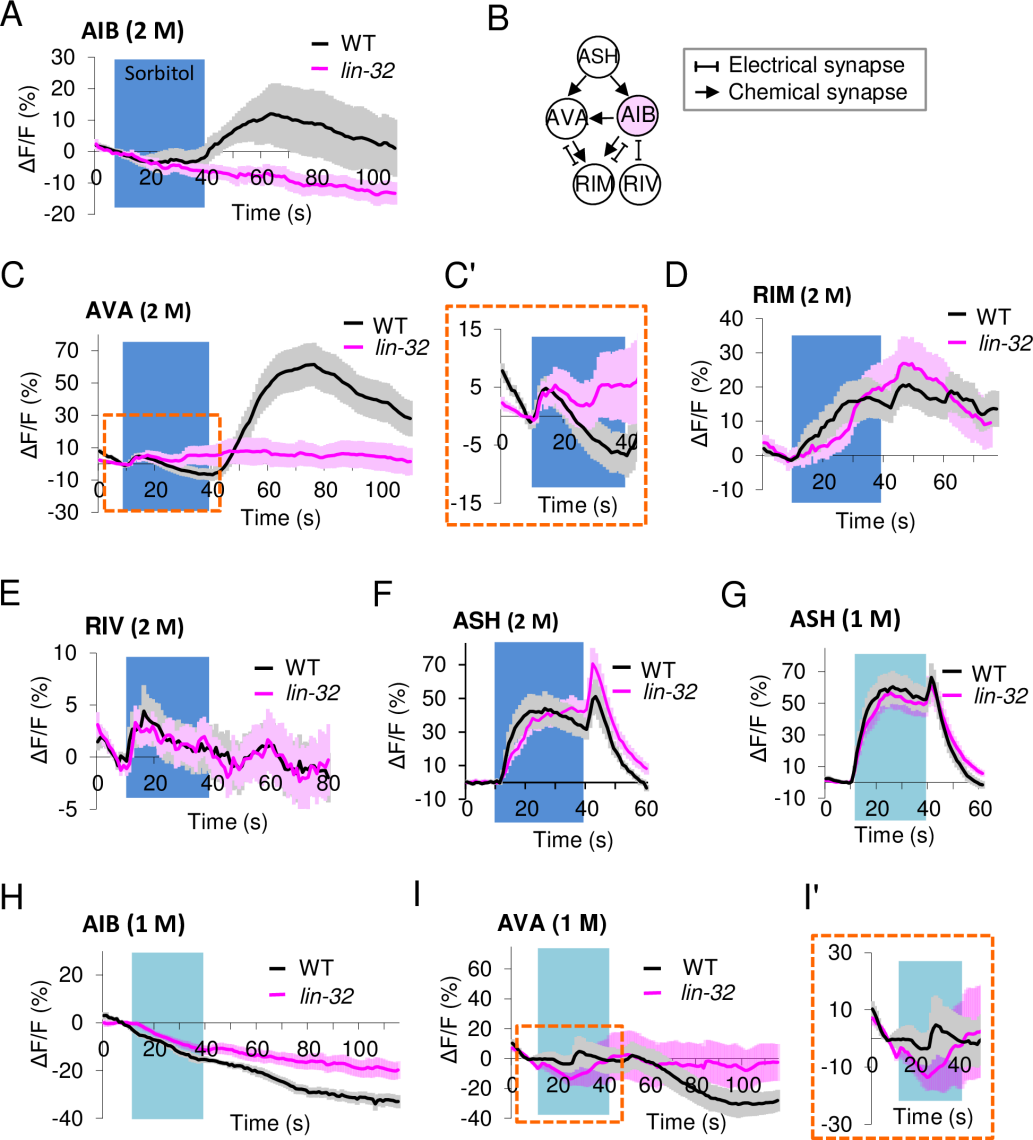
Strong stimulus-dependent OFF calcium increases that are present in AIB and AVA interneurons are absent in *lin-32* mutants. (A) Average calcium responses of AIB to 30-sec 2 M sorbitol stimuli under microfluidic chips. AIB OFF calcium increases occur in wild type animals but not in *lin-32* mutants. Wild type, n = 19; *lin-32* mutants, n = 22. (B) A schematic drawing of how AIB connects to three downstream neurons through chemical and/or electrical synapses. (C, C') Weak ON calcium increases occur in AVA in both animals (see an enlarged view at Fig 3C' shown by a dashed line), whereas strong OFF calcium increases occur only in wild type animals but not in *lin-32* mutants to 30-sec 2 M sorbitol. Wild type, n = 28; *lin-32* mutants, n = 35. (D-E) ON calcium increases in RIM (D) and RIV (E) occur in both animal types to 30-sec 2 M sorbitol. Wild type, n = 15 and 28, respectively; *lin-32* mutants, n = 17 and 33, respectively. (F) Both animals show normal biphasic intracellular calcium increase upon the introduction and removal of the 30-sec 2 M sorbitol stimulus (ON and OFF calcium increases). Wild type, n = 10; *lin-32* mutants, n = 16. (G) Both animals show short peak duration of intracellular calcium increase upon the 30-sec 1 M sorbitol stimulus. n = 20. (H) AIB does not respond to 30-sec 1M stimuli in either type of animal. Wild type, n = 22; *lin-32* mutants, n = 20, respectively. (I, I') OFF calcium increases do not occur in AVA in either type of animal upon the 30-sec 1 M sorbitol. An enlarged view at Fig 3I' shown by a dashed line. n = 21, 22. n means the number of individuals (animals). The data are presented as the mean ± SEM.

We performed calcium imaging experiments using GCaMP3 to examine the AIB interneuron response to an osmotic stimulus. In the wild type animals, we found an increase in the calcium concentration occurred at the time of removal of the sorbitol stimulus (the OFF calcium responses) (Fig 3A). Interestingly, in *lin-32* mutants expressing GCaMP3 in the AIB marker-positive cells, the OFF calcium increase was completely eliminated (Fig 3A).

Next, we attempted to analyze the AVA, RIM, and RIV neurons receiving inputs from ASH and AIB (Fig 3B). First, in the AVA interneurons of the wild type animals, both a weak ON calcium increase during stimulation and a strong OFF calcium increase after stimulation removal were observed (Fig 3C. Enlarged images of the area are indicated by the square at Fig 3C'). This result was similar to that of a previous study showing a weak and slow ON calcium increase during the stimulation of ChR2 in ASH neurons and a strong OFF calcium increase after stimulation [15]. The OFF calcium increase was selectively eliminated in the AVA interneurons of the *lin-32* mutants (Fig 3C), although normal differentiation of the AVA neuron was suggested (S2M Appendix, S3 Table).

We also analyzed the other downstream interneurons, namely, RIM and RIV (Fig 3B). The RIM response in the *lin-32* mutants was similar to that of the wild type (Fig 3D), and no statistically significant differences were detected (p > 0.05, ANOVA followed by the Tukey's post hoc tests). The RIV response was weak and the difference between both strains was also within the error bar (SEM) range (Fig 3E), suggesting a weak contribution to optimization of probability of omega turn. These ON calcium increase of the AVA and RIM neurons upon stimulus onset in the wild type animals were consistent with those in the previous study [11].

Finally, since the defect in AIB differentiation might lead to the remodeling of neural circuits at developmental stages and/or the response of sensory neurons might be modified by feedback through secretory factors [26, 27], we confirmed the response of ASH sensory neurons. Both animals showed strong biphasic ON and OFF calcium increases with 30 sec stimulation of 2 M and 1 M sorbitol solution (Fig 3F and 3G). In the wild type animals, duration of OFF-response peak were 4.05 ± 0.630 sec (means ± SEM) with 2 M sorbitol stimulus (n = 20) and 6.40 ± 0.859 sec with 1 M one (n = 20), showing significant difference between them (p < 0.05, Student’s *t*-tests). There is no significant difference between wild type animals and *lin-32* mutants with 2 M and 1 M condition (p > 0.05, Student’s *t*-tests). These results might suggest that the *lin-32* mutants have the processing defect in the central nervous system but not by a reduced sensation of sensory neurons, namely, "hypoesthesia".

Based on our observation, we hypothesized that the OFF calcium increases of AIB and AVA corresponded to strong stimuli and an increased omega turn frequency. When the stimulus was weakened to 1 M, the wild type animals showed no OFF calcium increases of AIB and AVA (Fig 3H and 3I), but might suggest stochastic weak ON calcium increases in AVA (Fig 3I, Enlarged images of the area are indicated by the square at Fig 3I'), like the *lin-32* animals.

**Fig 4:**
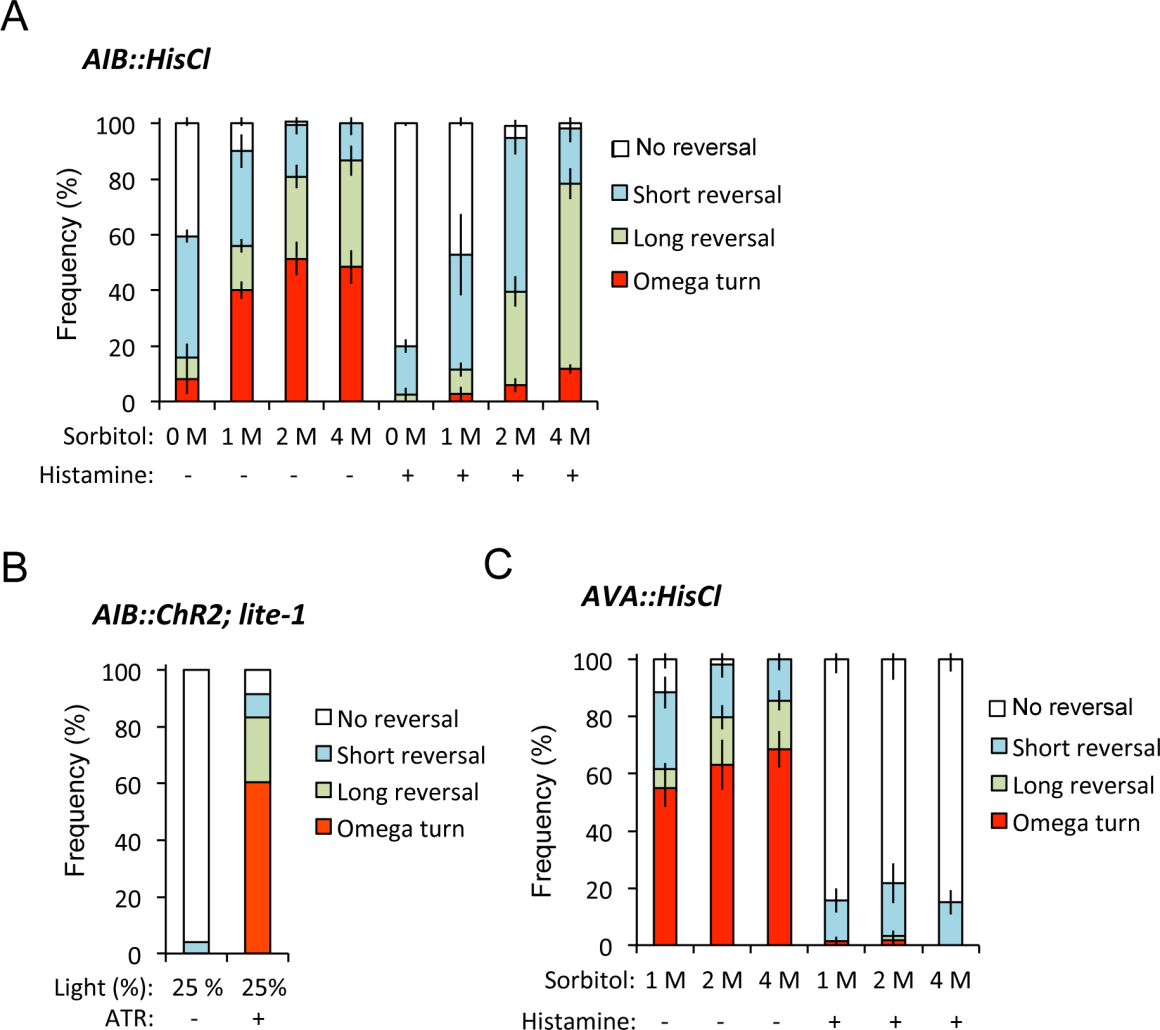
AIB calcium increases probability of omega turns. (A) AIB expressing HisCl1 (AIB::HisCl) inhibits omega turns in the presence of histamine. n = 4,6,16,7,4,5,16,5. n = plate (cohort) of approximately 10–20 animals. The data are presented as the mean ± SEM. (B) ChR2-induced excitation of AIB (AIB::ChR2(H134R)) increases omega turns during and after the 2-sec stimulation. n = 49, 57. n means the number of individuals (animals). (C) AVA expressing HisCl1 (AVA::HisCl) inhibits all avoidance behaviors in the presence of histamine. n = 6,6,7,7,6,6. n = plate (cohort) of approximately 10–20 animals. The data are presented as the mean ± SEM.

Next, to know whether decreased OFF calcium increases in AIB and AVA neurons suppressed omega turn, we used histamine-gated chloride channel HisCl1 to silence neurons [16]. We found that AIB silencing by application of histamine phenocopied the selectively impaired omega turns depending on proportional to stimulus intensity as observed in *lin-32* mutants (Figs 1F and 4A). This finding was consistent with the previous report demonstrating that AIB silencing reduced the omega turn frequency using a single glycerol concentration [16]. Conversely, ChR2-induced AIB excitation significantly drove omega turns with and without food (Fig 4B, S5A Appendix) (p < 0.001, Fisher's exact test). Thus, AIB seems to increase the relative frequencies of omega turns. On the other hand, AVA silencing reduced all types of avoidance behaviors in our experiments (Fig 4C), suggesting that AVA may simply determine whether animals escape rather than the optimization of behavioral types. Taken together, these data suggest that impaired optimization in *lin-32* mutants is presumably caused by the developmental and functional defects of AIB, which are the key neurons in optimization of probability of omega turns.

In our experiments, we found that ChR2-induced AIB excitation significantly increased omega turns without backward behavior (7 out of 30 individuals) compared to wild type (Ex-) animals (0 out of 30 individuals) (p < 0.01, Fisher's exact test). OFF-calcium increase occurs at the timing of removing the stimulus (Fig 3A), we hypothesized that *C*. *elegans* switched to omega turns after completely escaping from a noxious stimulus. Thus, we compared the behaviors of free-moving animals expressing ChR2 in ASH sensory neurons during and after blue-light irradiation (2 or 5 seconds). During the stimulation for 2 seconds, 93.8% of the animals continued backward movement throughout the stimulation (S5B Appendix), whereas 66.7% of the animals switched to omega turns within 1 second after the removal of stimulation following continuous backward behavior, regardless of the length of the stimulus (S5C Appendix). Conversely, when we used a longer stimulation for 5 seconds, 85.6% of the animals continued backward movement throughout the stimulation (S5B Appendix); however, 12.0% of the animals started omega turns during the stimulation, and 70.1% of the animals switched to omega turns within 1 second after the removal of stimulation (S5C Appendix). Our results suggest that the omega turn occurs in a safe place after escaping from a noxious stimulus by prompt backward movements.

### Electrical synapses via INX-1 are required for the optimization of probability of omega turn

**Fig 5:**
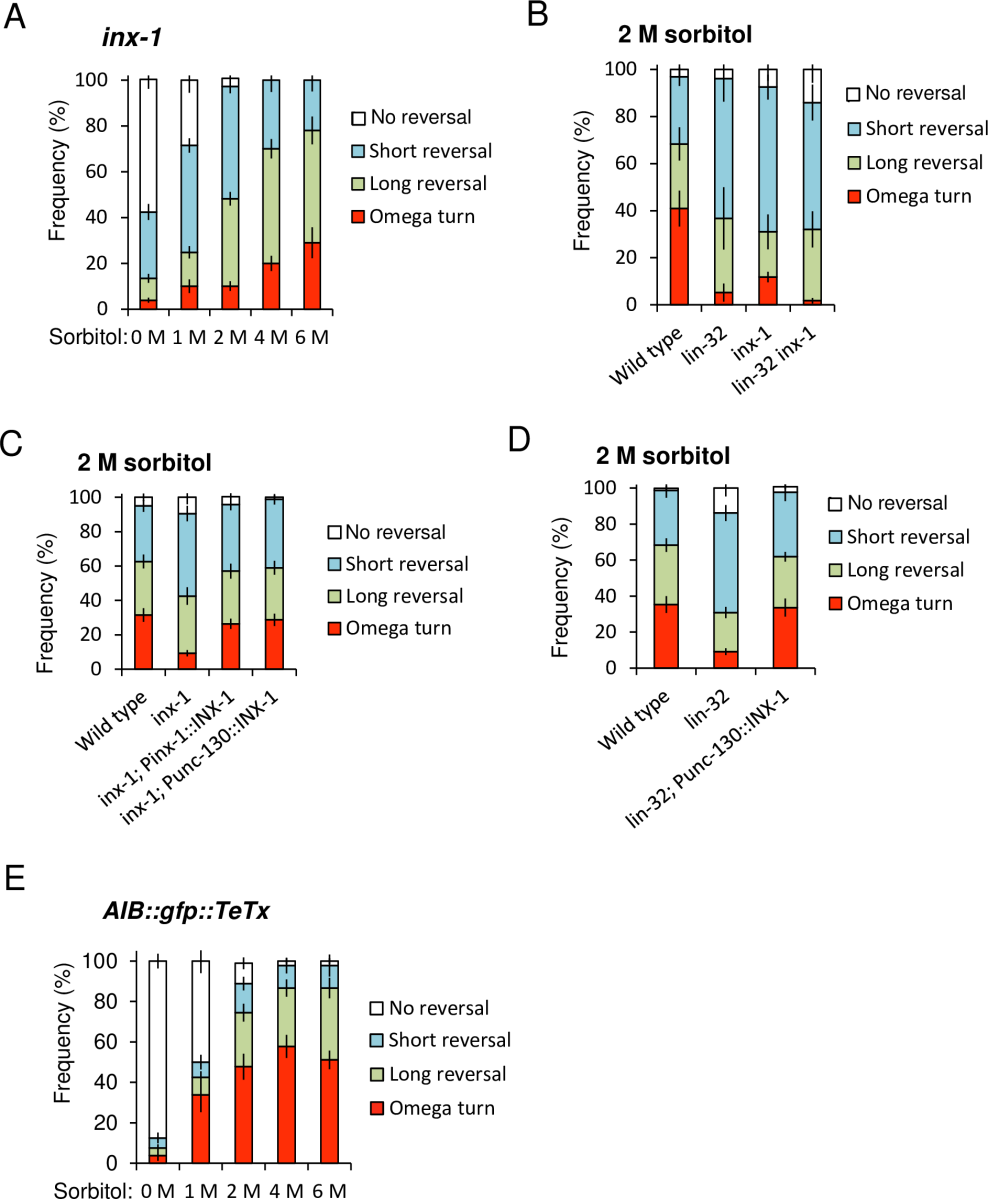
AIB electrical synapses are required for the optimization of probability of omega turn. (A) *inx-1* mutants show impaired avoidance behaviors independent on the sorbitol concentration. n = 33,10,35,10,10. (B) The frequency of avoidance behaviors toward 2 M sorbitol. *inx-1* mutants show reduced omega turns compared to the wild type animals. The *inx-1* phenotypes were not different from those of *lin-32* mutants and *lin-32 inx-1* double mutants. n = 4,4,4,12. (C) Expression of *inx-1* cDNA driven by its own promoter (*inx-1; Pinx-1*::*INX-1*) and the AIB lineage-selectively promoter (*inx-1*; *Punc-130*::*INX-1*) fully rescues osmotic avoidance defects in response to 2 M sorbitol. n = 21,21,22,13. (D) Expression of *Punc-130*::*INX-1* cDNA fully rescues osmotic avoidance defects in *lin-32* mutants in response to 2 M sorbitol. n = 21,21,13. (E) AIB::gfp::TeTx transgenic animals can adjust probability of omega turns depending on the sorbitol drop concentration. n = 8,8,9,9,9. n = plate (cohort) of approximately 10–20 animals. The data are presented as the mean ± SEM.

Almost all *lin-32* mutants failed to express *inx-1* (Fig 2C, S3 Table). INX-1 is the only known electrical synapse component of AIB interneurons [21]. To examine how AIB electrical synapses contributed to the optimization of avoidance behaviors, we analyzed the degrees of the behavioral defects of the *inx-1* null mutants. Compared to the wild type animals (Fig 1D), the *inx-1* mutants exhibited significantly reduced omega turns in 2 M, 4 M and 6 M sorbitol (Fig 5A) (p < 0.0001, ANOVA followed by the Tukey's post hoc tests). The defects in *inx-1* seemed to be slightly weaker than those of *lin-32*, but the differences were not significant between *inx-1* mutants and *lin-32* mutants or *lin-32 inx-1* double mutants (Fig 5B) (p > 0.05, respectively. ANOVA followed by the Tukey's post hoc tests). These behavioral data suggest *inx-1* is downstream of *lin-32*.

We performed AIB-specific INX-1 rescue experiments to verify that *inx-1* acted in AIB. The results showed that the expression of INX-1 cDNA under *Punc-130*, could fully rescue the defect, as could the authentic *inx-1* promoter (Fig 5C). In addition, INX-1 cDNA expression under the control of *Punc-130* also fully rescued the defects in the *lin-32* mutants (Fig 5D). In both cases, there were significant differences relative to each single mutant (p < 0.001, respectively. ANOVA followed by the Tukey's post hoc tests), and no significant differences relative to the wild type animals (p > 0.05, respectively. ANOVA followed by the Tukey's post hoc tests).

Expression of *inx-1* under the *Punc-130* promoter would also be expected to cause ectopic *inx-1* expression, and the potential consequences of this are unclear, for example, new electrical synapses might be generated, which could change the response to ASH stimulation. Then, to exclude transgene effects, we performed the drop test of wild type animals harboring *Punc-130*::*INX-1* transgene, and we found no effect (S5D Appendix) (p > 0.05, Fisher's exact test). Moreover, we also examined animals expressing an unrelated protein, mCherry, under the control of *Punc-130* as a control, and again with no effect (S5E Appendix) (p > 0.05, Fisher's exact test). These results indicate that the important role of *lin-32* in the synaptogenesis or synaptic functions of electrical synapses for behavioral optimization. AIB electrical synapses comprising *inx-1* are required for behavioral optimization depending on the stimulus strength. Impaired optimization of probability of omega turn in the *lin-32* mutants seems to be at least partly due to the disruption of electrical synapses comprising *inx-1* in AIB interneurons.

Our results suggest INX-1-containing gap junctions of AIB are required for determination of probability of omega turn. We tried to know the effect of disrupting synaptic transmission with tetanus toxin using transgenic AIB::TeTx strains [18]. Our created transgenic AIB::TeTx strain showed reduced frequency of reversal in free-moving condition as previously reported (S5F Appendix) (p < 0.001, Student's *t*-tests) [18], indicating AIB::TeTx works. It seemed that the frequency of turns slightly increases compared to wild type animals (Figs 1D and 5E), but there was no significant difference (p > 0.05, ANOVA followed by the Tukey's post hoc tests). This result strongly supports our hypothesis that AIB gap junction consisting of *inx-1* is important for determination of probability of omega turn, but chemical synapses have little effect on it.

Jang et al have described the use of *unc-1* dominant negative as transgenic disruptors of gap junction function [28]. To know whether expression of this gene phenocopies *inx-1* loss-of-function alleles, we analyzed their avoidance behaviors. However, there was no significant difference compared to wild type animals (S5G Appendix) (p > 0.05, ANOVA followed by the Tukey's post hoc tests). *unc-1* dominant negative might not be disruptor for INX-1 function.

### Omega turns are driven by strong neck contractions mediated by AIB interneurons

**Fig 6:**
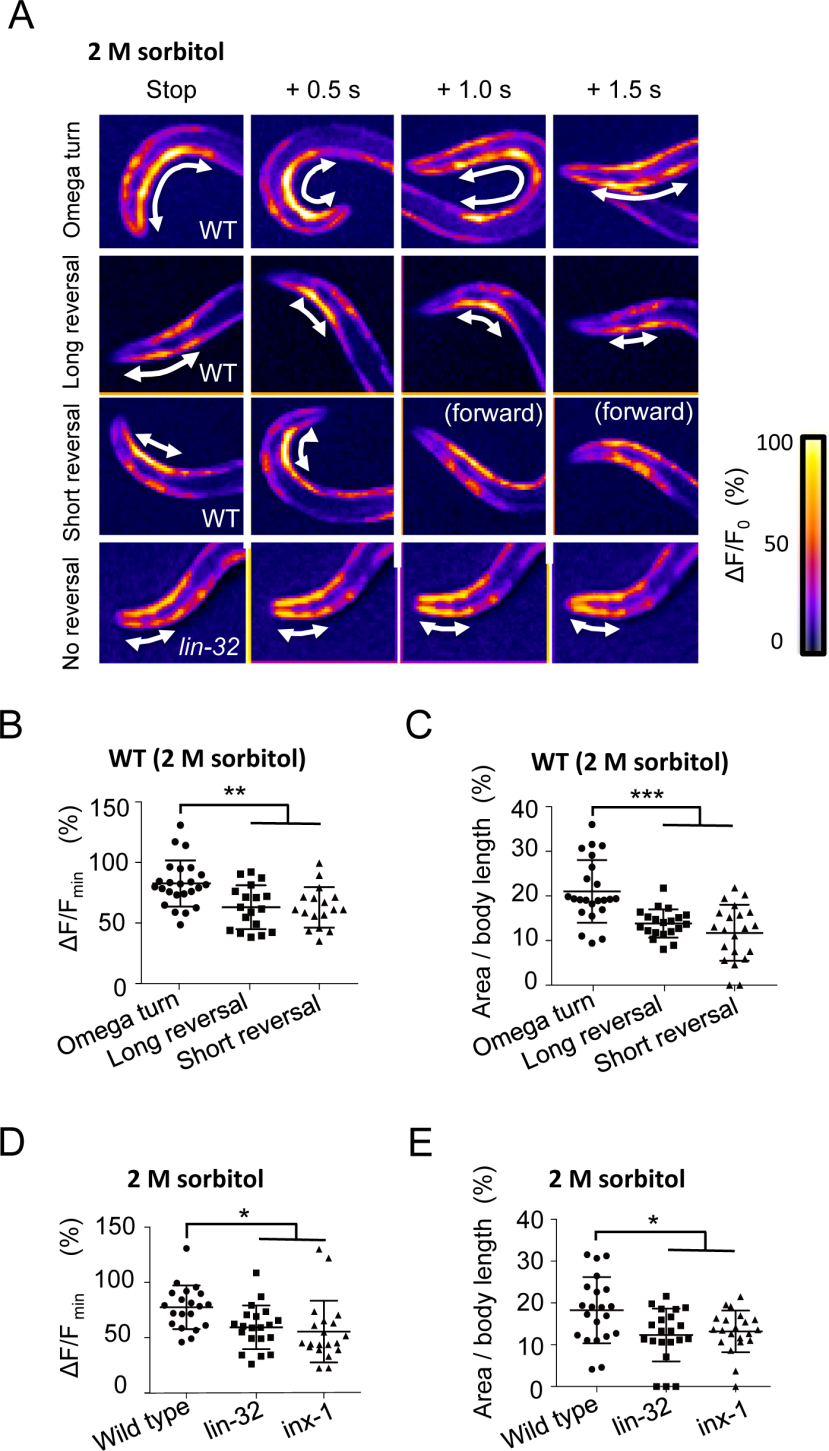
Omega turns are correlated with strong and broad calcium mobilization, and *lin-32* and *inx-1* mutants show impaired calcium mobilization. (A) Heat maps of calcium imaging of the neck muscles *in vivo*. Arrows indicate the area with high calcium. (B) Scatter plot of the maximum calcium fluorescence changes in individuals. The values are higher for omega turns than for reversals. **p < 0.01, ANOVA followed by the Tukey's post hoc tests. n = 24,18,17. (C) Scatter plot of the areas of the calcium increase in individuals. Areas are broader for omega turns than for reversals. ***p < 0.001, ANOVA followed by the Tukey's post hoc tests. n = 23,20,21. (D) Random samples of *lin-32* and *inx-1* mutants show tendency of impaired calcium fluorescence changes. *p < 0.05, ANOVA followed by the Tukey's post hoc tests. n = 20 in each strain. (E) Random samples of *lin-32* and *inx-1* mutants showed tendency of decreased calcium-induced areas. *p < 0.05, ANOVA followed by the Tukey's post hoc tests. n = 20 in each strain. n means the number of individuals (animals). Plots represent the mean ± SD.

AIB and AVA are known to directly or indirectly regulate neck muscle contractions via downstream RIA, RIV, and SMD neck inter-/motor neurons and DA/VA motor neurons [10,12]. To investigate the relationships between muscle contraction and the avoidance behaviors, we analyzed the calcium concentrations in the neck and body wall muscles during the presentation of avoidance behaviors in free-moving *C*. *elegans*, which expresses GCaMP2 in the body wall muscles. The neck-bending step seemed to be critical for the omega turn, so we focused on the events for a short period after the backward movement had stopped. In a wild type animal exhibiting an omega turn, a strong calcium mobilization occurred in the ventral neck muscles at the beginning of the omega turn, and the mobilization propagated back to the body wall muscles (Fig 6A). In the long and short reversals, the calcium mobilization remained weak and narrow (Fig 6A). We quantified the peak calcium concentrations in the neck muscles and the area of the calcium increase per the whole body length. The results showed that both peaks and areas were significantly higher in the omega turns than in the short and long reversals (Fig 6B and 6C) (p < 0.01 and 0.001, respectively. ANOVA followed by the Tukey's post hoc tests). Both the random sampling *lin-32* and *inx-1* mutants showed their peak calcium concentrations and area significantly varied to low values compared with that of wild type animals (Fig 6D and 6E) (p < 0.05, respectively. ANOVA followed by the Tukey's post hoc tests). The results suggest that both mutants fail to perform strong neck muscle contractions that are associated with omega turn.

## Discussion

### A hypothetical circuit model for optimization of avoidance behaviors depending on stimulus intensity

**Fig 7:**
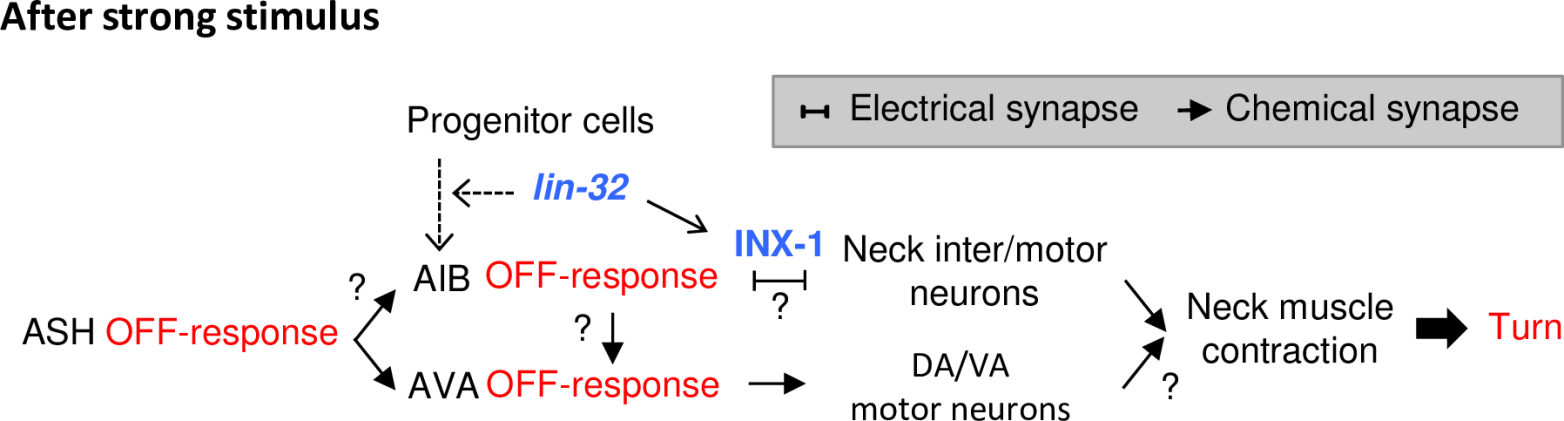
A hypothetical model for the optimization of avoidance behaviors. A schematic model illustrating the neural circuit for the optimization of omega turns and reversals. The dotted arrows indicate the developmental step promoted by *lin-32*.

In the present study, we showed that *lin-32* was required for AIB differentiation and used a hypothetical neural model to show that AIB OFF calcium increase are key to optimizing both the type and timing of distinct omega turn depending on the intensity of the stimulus (Fig 7). Our study provides the first observation of the neural response of AIB in nematodes exposed to noxious stimuli for a long time. Under the free-moving condition, animals quickly escape from the stimulus source, so the ON and OFF calcium increase had not been able to be separated. The probability of the omega turn is characterized by the difference in terms of body bending and final destination. This dynamics appears different from those of simple reflexes, such as touch responses [2,3,4,5,6].

Typically, avoidance behavior consists of four steps: backward behavior, stopping, bending of the neck to alter the direction, and a return to forward movement [10]. The reversal occurs during the ON calcium increase in AVA. OFF calcium increase in AIB is observed after the removal of strong stimulation to ASH appears highly correlated with the phenomenon.

The OFF calcium response in AIB can transmit the information directly or indirectly to the neck inter/motor neurons via electrical synapses comprising INX-1 (Fig 7). The OFF calcium increase in AIB might also affect the OFF calcium increase in AVA via chemical synapses. In the previous study, *eat-4* mutants showed a partially degraded OFF calcium increase only but not an ON calcium increase, suggesting the involvement of chemical synapse for the OFF calcium increase [15]. Our hypothesis is also consistent with the observation that the initiation of the OFF calcium increases in AVA lagged behind that in AIB (Fig 3A and 3C). The strong contraction of the neck muscles correlates with the induction of "omega bends" in the omega turn (Fig 6B and 6C). Both circuits ultimately coordinate the neck bends.

Our model that is based on the concept of the OFF calcium increase is presumably consistent with the observation that the majority of the free-moving animals exhibit an omega turn immediately after the removal of stimulation (S5C Appendix). We are unclear as to how AIB is inhibited during the stimulation while being excited after the stimulus, although ASH is excited in response to a noxious stimulus and a direct connection between ASH and AIB is present [12]. OFF responses tend to occur after inhibition because channels are released from inactivation during hyperpolarization [29]. AIA is known to receive synapses from ASH and send outputs to AIB [12]. Given that AIA or other neurons inhibit AIB at the early phase of the noxious stimulus and that the strong OFF responses of ASH directly stimulate AIB after the stimulus (Fig 3F), AIB may also be strongly excited.

Whatever the cellular mechanism, ON- and OFF-responding neural circuits might explain the biological implication of a survival strategy whereby *C*. *elegans* immediately exhibits an initial backward behavior as an emergency response upon exposure to a fatal environment and then performs a relatively time-consuming omega turn in a safe place after completely escaping from the stimulus. An OFF calcium increase has also been observed in the central nervous systems of mammals [30], possibly predicting a common neural mechanism that regulates the timing of recovery behaviors in many species.

We have stimulated the animals with 2 M for 30 secs to mimic strong stimulation (Fig 3A–3F). A similar biphasic ASH response occurs with the high concentration of CuSO_4_ as noxious stimuli [31], supporting the notion that our stimulus conditions can mimic strong noxious stimuli. However, we should note that the delay for detection of calcium response using GCaMPs tends to be longer than that for the behavioral response (Fig 3, S5C Appendix) [15]. Ultimately, an observation of free-moving animals after more than 30 seconds of stimulation will likely provide a strict conclusion regarding the timing of the omega turn.

The deficiency in *lin-32* induced AIB differentiation defects, and the impaired AIB neural responses correlated with weak neck muscle contractions and impaired omega turns, even with a strong stimulus (Fig 6A, 6D and 6E). These results indicated the important role of *lin-32* for AIB function and the optimization of probability of omega turn. Whether the majority of *lin-32* mutants lost AIB cells remains unclear; however, synaptic and/or intracellular functions are certainly disrupted even when AIB marker-positive cells are present (S4 Appendix). Strangely, there was no noticeable change of calcium response in the downstream RIM interneurons in the *lin-32* mutants (Fig 3D). We speculate that this is because of the normal ON calcium increase of upstream AIB and AVA (Fig 3A and 3C), and/or complementary other unknown neuron. Further experiments are necessary to elucidate the question.

### Electrical synapses in avoidance circuits

We have demonstrated that AIB electrical synapses play a major role in the optimization of avoidance behaviors. In recent years, the importance of not only chemical synapses but also electrical synapses for avoidance behaviors has been unveiled [8]. However, the understanding of the relationships among the molecular players of circuit formation, response patterns of neurons and muscles, and principle of the behavioral choice depending on the strength of stimulus is poorly elucidated. Additionally, in vertebrates, electrical synapses are most commonly found in avoidance reflex circuits [32,33]. In *C*. *elegans*, electrical synapses in AIA interneurons, which are modulator neurons of AIB, can also modulate ASH sensitivity through a feedback loop [8]. The secondary circuit might allow for a rapid behavioral switch by electrical synapses.

The defects in *inx-1* mutants tend to be slightly weaker than those of *lin-32* (Fig 5B). *lin-32* may be also involved in other genes expression in AIB. Although we think that reduced omega turn of *lin-32* mutants is mainly caused by reduced *inx-1*, other genes in AIB might be also involved in AIB functions. The main AIB chemical synapses *glr-1* mutants often failed to avoid osmotic stimuli [34]. *glr-1* expression was also impaired in *lin-32* (S3 Table), we had tried to examine the effects of *glr-1* mutation on the optimization of avoidance. However, because of the more pleiotropic expression of the *glr-1* gene, we could not examine the pattern changes of the avoidance behaviors.

### *atonal* determines AIB fate in the avoidance circuits

To the best of our knowledge, our report is the first to address the function of the proneural gene *atonal* and its orthologs in the optimization of avoidance behaviors. In development, proneural genes regulate the expression of downstream transcription factors, and their combination determines intrinsic neural identity and the specificity of synaptic connections [20,24]. Genetic analyses of human fetuses has revealed that the human homolog *ATOH1* is expressed in nociceptive circuits, including the spinal cord, cerebellum, and prefrontal cortex [35,36], presumably implying the contribution of *ATOH1* to the differentiation of the nociceptive pathway for the optimization of avoidance behaviors [37,38,39]. In addition, there is a common feature that *atonal* contributes to the differentiation of glutamatergic neurons in both mammals and *C*. *elegans* [40]. Although functions for avoidance behavior are unclear in mammals because the null mutation causes a lethal phenotype [41], our results imply a conserved role for avoidance behaviors. In addition, involvement of *atonal* in electrical synapses had been unknown in both mammals and *C*. *elegans*. The hypothetical *atoh1* downstream genes that have been identified by transcriptome analysis using conditional KO mice do not include electrical synapse genes [42,43,44]. Mammalian *atoh1* may not contribute to electrical synapses or might act in restricted brain areas.

By RNAi screening, we have obtained 18 additional candidates associated with abnormal avoidance behaviors (S2 Table). Interestingly, some subsets are included in the same genetic cascades: *lsy-2*, *fozi-1* and *die-1* [45] and *ceh-10* and *ceh-23* [46]. In addition, *nob-1* and *php-3* are duplicated genes [47]. These genes might also act together in the formation or function of the avoidance circuit. In the near future, further analyses of the additional 18 candidate genes and additional large-scale transcriptome analyses will hopefully clarify the entire gene network of the neural circuit construction for the optimization of avoidance behaviors.

## Materials and methods

### Nematode deletion mutants and culture

*C*. *elegans* strains were cultured using standard techniques [48]. All mutants were backcrossed at least twice with N2.

Strains carrying following mutations were obtained from the trimethylpsoralen/ultraviolet (TMP/UV) mutagenized library, as described previously [49] and identified by PCR amplification with primers spanning the deletion regions: *lsy-2(tm5748)*, *lin-11(tm5323)*, *ham-2(tm5905)*, *nob-1(tm4459)*, *unc-130(tm5323)*, and *unc-9 (tm5479)*. Primers used for nested PCR screening were as follows; *tm5748*_1st round: TCTGCACTTGGCGGCAAGGT, ATTAGCAGAGGAGAGTCGAC, 2nd round: TCTAGCAGTCTCGAGCTGTT, CGTTTTCGCAACCCGCCACA, *tm5323*_1st round: ACTGCGAATCGTCACCCACT, GGGACACATTCAAGAGGTGT, 2nd round: TCACCCACTCCTGGATATAT, GACGTGAGAAAATAGCGACT, *tm5905*_1st round: GGGGGAAGCGGCTGTTTTAG, GACTAGACGATCCACCACTG, 2nd round: GTTGTAGATAGCACATCCGA, TCAATGGTGGTTCGCCCTGA, *tm4459*_1st round: GCGAGGCGAAATTTATGGGT, AGTTAGTATGGCCCCCCATC, 2nd round: TGGGGGAGGTCTCAACGGTA, AGTGAGGGGGGCCGTACTAA, *tm5323*_1st round: ACTGCGAATCGTCACCCACT, GGGACACATTCAAGAGGTGT, 2nd round: TCACCCACTCCTGGATATAT, GACGTGAGAAAATAGCGACT, *tm5479_*1st round: CAGTTATCAATGATCGGGGT, CCTATTGTATGACTTCGCGA, 2nd round: GGGTACACCTATTCTAGAGA, CAATTCGTGAAGAAGGGTGC.

*inx-1(tm9620); zwIs132[myo-3p*::*GCamp2 + lin-15(+)]* was obtained using the CRISPR/Cas9 system [49, 50]. For generating *inx-1* deletion mutants, the multi-guide RNA plasmid was obtained by PCR using primers containing two gRNA sequences (CATGTGTGTCCAGAAGCGATGGG GGTCGTTATGCGTTTCCAGGGGG) designed using CHOPCHOP Harvard (https://chopchop.rc.fas.harvard.edu/) [51] and pDD162 plasmid. PCR primers for screening were TGTGTTCGGAACGCAGCGTG and GCAACGAGGAAACTGGGATCCAAACG. N2 Bristol, *lite-1(ce314)*, *fax-1(ok624)*, *osm-6 (p811)*, *unc-86(e1416)*, *mec-3(gk1126)*, *ceh-17(np1)*, *ceh-23(ms23)*, *php-3(ok919)*, *xnd-1(ok708)*, *xnd-1(ok709)*, and *nhr-185(gk957)* were obtained from *Caenorhabditis* Genetics Center.

### Plasmid construction

For optogenetics, pPD_*Psra-6*::*ChR2(H134R)*::*mCherry* and pPD_*Pinx-1*::*ChR2(H134R)*::*mCherry* were constructed by subcloning the 2.4 kbp and 1 kbp fragments of the upstream genomic region of the *sra-6* and *inx-1* genes, respectively.

For RNAi screening, plasmids were constructed by subcloning using primers as follows; L4440_*syd-9*: cggtatcgataagcttTCAATATACGTTAGTGCAGATGGCA, cggtatcgataagcttTCAATATACGTTAGTGCAGATGGCA, L4440_*R03E1*.*4*: ggagaccggcagatctAGCCGAGTTAGGGCTTCTGAAG, cggtatcgataagcttCTACGAGAGAATATTGTCAATTGTGAAGG, L4440_*sir-2*.*4*: ggagaccggcagatctATGAAATCTGCAAAATACAAAACCGTTTC, cggtatcgataagcttTTAGCTAATTTTCAATGGAATAGGCACT, L4440_*nhr-52*: ggagaccggcagatctATGAAATGTTTAGTTTGTTGTTCGTATGC, cggtatcgataagcttCTCAGGAACCATACATTGAAACATC, L4440_*F26H9*.*2*: ggagaccggcagatctATGACGTCAAATGAAGATGTGAGCAG, cggtatcgataagcttCATCAGTTGAAGATCCAGGTGGTG, L4440_*lin-22*: GGAGACCGGCagatctATGACGTCATTCCTGTGCTCCG, cggtatcgataagcttCTACACTCTACTTCGATCCTC, L4440_*Y47G6A*.*7*: ggagaccggcagatctATGAGATTCCCGTCTACATTCTCC, cggtatcgataagcttTTAAGCGTCTGGATCTTCTGTTGC, L4440_*cog-1*: ggagaccggcagatctATGAGCCTAATCATGTCCGAACCG, cggtatcgataagcttCTAAATTCCAGCGCTGAGAAATGGG, L4440_*ceh-9*: ggagaccggcagatctATGGAAACTGACTTGCTCTTCCAAC, cggtatcgataagcttTTATTGCGGCTTCACAATCTGATCATC, L4440_*hlh-17*: ggagaccggcagatctATGGAGAAAATTGGAATTGATACTGCTTTC, cggtatcgataagcttTCAGTTTGCATCAGGCTTTAAGGATTTG, L4440_*ceh-38*: ggagaccggcagatctATGGAGTCATCACGGACCGC, cggtatcgataagcttAAAGAGTTCCCTGACTACGACAC, L4440_*lin-13*: ggagaccggcagatctATGGATGAGTTTGAGCTGTTTCAAC, cggtatcgataagcttCACAGCAATTTGGGCGAACAGTTG, L4440_*C50H2*.*6*: ggagaccggcagatctATGGCTTCACTTGCAAAAAGTATTGTG, cggtatcgataagcttTTATTTGCTGCTCATCTTCATTGAGTCG, L4440_*tbx-37*: ggagaccggcagatctATGTACTCGTGCACCTCTCCTTC, cggtatcgataagcttCCATTATTGAGCATTTGTGGTTGTCC, l4440_*ceh-30*: ggagaccggcagatctATGTCACTTCTCGACCCTCGG, cggtatcgataagcttCTATTCTGAGTTGCTGGAAACATCC, L4440_*lin-36*: ggagaccggcagatctATGTCGGAAGAATTGCTGAGTACTAG, cggtatcgataagcttGGCCAGCATTCAAGTGACGACG, L4440_*fkh-9*: ggagaccggcagatctATGTGTAGTACAATGACAGCAACC, cggtatcgataagcttCTATTGAAGAAGAAAAGACTGGCTC, L4440_*nhr-40*: ggagaccggcagatctCCCGGAAGGAACACTTTGCG, cggtatcgataagcttCTACAAGCCAACGATAATGAGCTG, L4440_*hlh-14*: ggagaccggcagatctTGGGAAACATCTCCATGAGG, cggtatcgataagcttTGCCAATATCCACCCTCCTA, L4440_*C02F12*.*10*: ggagaccggcagatctATGACGTCTAAAACGGTAAGTTTGAA, cggtatcgataagcttTCAAACATTGTTCTGTTGATCAGAAGG, L4440_*dmd-8*: ggagaccggcagatctATGTCCTCATCTTCCCTGTCATCTTC, cggtatcgataagcttTCAATCGGTTTCAGCGCAGCTAATTG, L4440_*F34D6*.*2*: ggagaccggcagatctATGAGTAGAAATCAGCTTCTGGAGTC, cggtatcgataagcttCCTCGGAAAATTCTGAGAAAGTATATT, L4440_*hlh-30*: ggagaccggcagatctGCCGATGACGACGACCGC, cggtatcgataagcttTTACGAAAAGTCCATGTGATAATGACCCG, L4440_*hlh-32*: ggagaccggcagatctGAGAAAATGAGCAGCGATCTAAC, cggtatcgataagcttCTAATCTCCTTCGGATGGTGTTG, L4440_*lin-15B*: ggagaccggcagatctCCGCAACACTGTGCAGTGCT, cggtatcgataagcttCCAAACAAGACATCTTGTGAGGAA, L4440_*mab-23*: ggagaccggcagatctATGCTCTTTATGTGCATTAAATACCAAAC, cggtatcgataagcttCTAGATCAATGACGTGGCAGATTCTC, L4440_*plp-1*: ggagaccggcagatctATGTCGGACGGAAGTGTTGAAAGG, cggtatcgataagcttCTACTTTCCTTGGTTGGCGATGATG, L4440_*rabs-5*: ggagaccggcagatctATGATTGGAGCAACTGGTTCAGGTG, cggtatcgataagcttGGTGACAACTCGAACATAAGTTGGC, L4440_*tag-233*: ggagaccggcagatctATGAATGGCGACACGGAAGTGC, cggtatcgataagcttGATGGCTTCTGCAATTGTAACTCC, L4440_*ZK757*.*4b*: ggagaccggcagatctATGTCTTGTCGGGATTGCACAAGG, cggtatcgataagcttCGAACATTTAGATCTTCTTCGTCC.

For the *lin-32* rescue experiment, pFX_*Plin-32*::*LIN-32* was constructed by cloning the 5.1 kbp genomic fragment (from 4.2 kbp of the genomic sequence upstream of the mature *lin-32* sequence), which was amplified by PCR from genomic DNA. pFX_P*unc-130*::*LIN-32*, the cDNA clone encoding *lin-32* (878 bp) was obtained by reverse transcription (RT)-PCR from mixed-stage *C*. *elegans* Bristol N2 cDNA and cloned into the pFX vector with 5.9 kbp of the upstream genomic region of the *unc-130* gene.

For the analysis of cell differentiation, to generate the pPD_*Pinx-1(1k + 50 bp)*::*Venus* plasmid, 1 kbp of the upstream genomic region and the 50 bp coding region of the *inx-1* gene were cloned into the 5’ region of Venus containing the original *gfp* of the pPD95.75 vector in-frame. To generate the pPD_*Pops-1*::*gfp*, *and* pPD_*Pinx-1*::*gfp* plasmids, 2.2 kbp or 1 kbp of the upstream genomic regions of the genes, as the promoters, were cloned into the 5’ region of *gfp* in-frame. To generate the pFX_*Pnmr-1*::*mCherry* and pFX_*Podr-2*::*DsRedx* plasmids, 5 kbp or 2.7 kbp of the upstream genomic regions, including the ATG codons of the genes, were cloned into the 5’ regions of the pFX_*mCherry* and pFX_*DsRedx* vectors in-frame.

For calcium imaging, pPD_*Pinx-1*::*G-CaMP3* was constructed by subcloning *G-CaMP3* sequences into the pPD_*Pinx-1*::*Venus* vector instead of *Venus*. pPD_*Pnpr-4*::*G-CaMP6s* was cloned into the pPD95.75 vector with 4.4 kbp of the upstream genomic region of the *npr-4* gene and the *G-CaMP6* sequences instead of *gfp*.

For the *inx-1* rescue experiment, to generate the pPD_*Pinx-1*::*INX-1* cDNA and *Punc-130*:: *INX-1* cDNA, the cDNA clone encoding *inx-1* (1287 bp) was obtained by RT-PCR from mixed-stage *C*. *elegans* Bristol N2 cDNA and cloned into the pPD vector with 1 kbp or 5.9 kbp of the upstream genomic regions of the *inx-1* and *unc-130* genes, respectively. All coding sequences used in the present study were verified by sequencing.

All plasmids were constructed using In-Fusion HD Cloning Plus (Takara Bio USA, 638909). pPD95.75 was a gift from Dr. Andrew Fire. pcDNA3.1/hChR2(H134R)-mCherry (Plasmid #20938), L4440 (Plasmid #1654), G-CaMP3 (Plasmid #22692), pGP-CMV-GCaMP6s (Plasmid #40753), and pDD162 (*Peft-3*::*Cas9* + Empty sgRNA) (Plasmid #47549) were obtained from Addgene (www.addgene.org).

### Transgenic lines and strains

To generate the *tmEx2531* transgenic animals and *tmIs825* (Fig 1B, 1C and 1E, S1 and S2 Tables, S1A and S1D Appendix, S5B and S5C Appendix, S1 and S2 Movies), pPD_*Psra-6*::*ChR2(H134R)*::*mCherry* (150 ng/μl) was injected with *Pges-1*::*EGFP* as an injection marker (50 ng/μl) into *ce314(lite-1)*, and the extrachromosomal array was integrated into the genome by using UV/TMP chromosomal integration methods [47].

To generate the *tmEx3738* transgenic animals (Fig 2C), pPD_*Pinx-1*(1k promoter and 50 bp of the cording region)::*Venus* (100 ng/μl) was injected with *lin-44p*::*gfp* (a gift from Y. Iino, University of Tokyo) (100 ng/μl) as an injection marker together into *hdIs32*. To generate the *tmEx3143* transgenic animals (Fig 2D), pFX_*Plin-32*::*LIN-32* (20 ng/μl), pFX_*Pnmr-1*::*mCherry* (100 ng/μl), and pBluescript (80 ng/μl) were co-injected into *tm2044(lin-32); tmIs825*. To generate the *tmEx3902* transgenic animals (Fig 2D), pPD_*Punc-130*::*LIN-32* cDNA (20 ng/μl), pFX_*Podr-2*::*DsRedx* (80 ng/μl), pPD_*Pinx-1*::*gfp* (80 ng/μl) and pBluescript (20 ng/μl) were co-injected into *lin-32(tm2044); tmIs825*. To generate the *tmEx3905* transgenic animals as a control (S4 Appendix), pFX_*Podr-2*::*DsRedx* (80 ng/μl), pPD_*Pinx-1*::*gfp* (80 ng/μl) and pBluescript (40 ng/μl) were co-injected into *lin-32*(*tm2044*).

To generate the *tmEx3363* transgenic animals (S2I–S2K Appendix), pFX_*Pgcy-28(d)*::*EGFP* (133 ng/μl) and pFX_*Punc-122*::*mCherry* (67 ng/μl) were co-injected into N2. To generate the *tmEx3829* transgenic animals (S3G and S3H Appendix), pPD_*Pops-1*::*gfp* (90 ng/μl) and pFX_*Podr-2*::*DsRedx* (90 ng/μl) were injected with *lin-44p*::*gfp* (20 ng/μl) as an injection marker into N2.

To generate the *tmEx3866* transgenic animals (Fig 3A and 3H), pPD_*Pinx-1*::*G-CaMP3* (180 ng/μl) and pFX_*Podr-2*::*DsRedx* (20 ng/μl) were co-injected into N2. To generate the *tmEx4532* transgenic animals (Fig 3C, 3C', 3E, 3I and 3I'), pPD_*Pnpr-4*::*G-CaMP6s* (160 ng/μl), pFX_*Pnmr-1*::*mCherry* (20 ng/μl) and *lin-44p*::*gfp* (20 ng/μl) were co-injected into N2.

To generate the *tmEx4456; ce314* transgenic animals (Fig 4B, S5A Appendix), pPD_*Pinx-1*::*ChR2(H134R)*::*mCherry* (180 ng/μl) was injected with *lin-44p*::*gfp* (20 ng/μl) as an injection marker into *lite-1(ce314)*.

To generate the *tmEx4573* (Fig 5C), pPD_*Pinx-1*:: *INX-1* cDNA (20 ng/μl), *Pnpr-9*::*gfp* (80 ng/μl), pBluescript (100 ng/μl), and *Punc-122*::*mCherry* (100 ng/μl) were co-injected into *inx-*1(tm3524). To generate the *tmEx4724* transgenic animals (Fig 5C), pPD_*Punc-130*:: *INX-1* cDNA (20 ng/μl), *Pnpr-9*::*gfp* (80 ng/μl), pBluescript (80 ng/μl), and *lin-44p*::*gfp* (20 ng/μl) were co-injected into *inx-1(tm3524)*. *tmEx4724* was crossed with *lin-32(tm2044)* (Fig 5D). To generate the *tmEx5108* transgenic animals as a control (S5E Appendix), pPD_*Punc-130*:: *mCherry* (20 ng/μl), pBluescript (80 ng/μl), pPD_*Pinx-1*::*gfp* (20 ng/μl), and pFx_*Podr-2*::*DsRedx* (80 ng/μl) were co-injected into N2.

To generate the *peEx2742* transgenic animals (Fig 5E, S5F Appendix), *npr-9p*:: *gfp*::*TeTx* (100 ng/μl) and *unc-122p*::mCherry (100 ng/μl) were co-injected into N2. To generate the *tmEx5110* transgenic animals (S5G Appendix), *npr-9p*:: *UNC-1a(n494)*::*SL2*::*EGFP* (30 ng/μl) [28], pBluescript (150 ng/μl), and *Plin-44*::*gfp* (20 ng/μl) were co-injected into N2.

*kyIs620 [inx-1*::*HisCl1*::*sl2*::*GFP + myo-3*::*mCherry]* and *kyEx4863 [rig-3*::*HisCl1*::*SL2*::*mCherry]* (Fig 4A and 4C) were gifts from Dr. Cornelia I. Bargmann, and *wdIs3* [*del-1*::*GFP + dpy-20(+)]; dpy-20(e1282)* (S2O–S2Q Appendix) was a gift from Dr. David Miller. *lin-15B(n744); uIs57 [unc-119p*::*YFP + unc-119p*::*sid-1 + mec-6p*::*mec-6]* (S1 and S2 Tables), *kyIs39[sra-6*::*GFP + lin-15(+)]* (S2A–S2C, S2G and S2H Appendix, S3 Table), *otIs292 [eat-4*::*mCherry]* (S2G and S2H Appendix, S3 Table), *dpy-20(e1282); ksIs2 [pdaf-7*::*GFP + rol-6(su1006)]* (S3C and S3D Appendix), *lin-15B(n765); kyIs37 [odr-10*::*GFP + lin-15(+)]* (S3E and S3F Appendix), *hdIs32[glr-1*::*DsRed2]* (S2L–S2N Appendix, S3 Table), *akIs3[nmr-1*::*GFP + lin-15(+)]* (S2L–S2N Appendix, S3 Table), *sraEx80 [sra-6p*::*chop-2(H134R)*::*mCherry; osm-10p*::*G-CaMP; unc-122p*::*mCherry]* (Fig 3F and 3G), and *zwIs132 [myo-3p*::*GCamp2 + lin-15(+)]* (Fig 6) were obtained from *Caenorhabditis* Genetics Center.

### ChR2(H134R)-induced avoidance assay

Seventy microliters of OP50 culture medium containing 2 μl of 100 mM all-trans retinal (ATR) (Sigma-Aldrich, R2500) was placed on a NGM plate and incubated for 0.5–16 h at room temperature. Then 10–30 adult animals were placed on the plates for 2–48 h in the dark [15]. The animals were individually irradiated with 12.5%, 25%, or 100% blue light (0.200, 0.395, and 1.58 μw/cm^2^, respectively) using CFP (365 nm) and ND filters at their heads for 2-sec. We observed their behaviors beginning during 2-sec light stimulations until the animals resumed forward movement. We classified the types of avoidance behaviors of approximately 10 animals per plate and calculated the percentage of each behavior. We performed the experiments on at least three different days and calculated the average percentage. The number for each analyzed plates is described in the figure legends as "n = x".

### Drop test

Animal preparation for the drop test was performed using the standard protocol [13]. We placed a small droplet of 0–6 M sorbitol or 0–4 M glycerol dissolved in S basal in the direction of the forward movement of the nematode using a 1 x 90-mm glass capillary with filament (Narishige Scientific Instrument Lab, GD-1). The nematode touched the droplet with only the tip of its nose. We omitted the data for animals soaked in the droplet. Responses were classified as omega turns, long reversals or short reversals, as previously described [10]. Short and long reversal are subdivided by the backward length with 1~2 head swings (but not "body bends") or three head swings or greater without omega turn, respectively. An omega turn is specifically defined by reorientation angles (θ ≧135) following the backward behaviors with/without either short reversal or long reversal. Each score was calculated for approximately 10 animals. We performed the experiments on at least three different days and calculated the average percentage. The number for each analyzed plates is described in the figure legends as "n = x". Note that we always checked the score of the wild type animals in the beginning and ensured that the scores were typically because the dry condition of the plates sometimes affected the score. If necessary, we adjusted the amount of droplets. Then, the experimenter began the experiments of the nematode strains in a blinded condition.

For rescue experiments (Figs 2D, 5C and 5D), we selected *Pinx-1*::*Venus* marker-positive animals as AIB-rescued animals since *Ex* led to a mosaic expression pattern. We classified the types of avoidance behaviors of approximately 10 animals per plate and calculated the percentage of each behavior. We performed the experiments on at least three different days and calculated the average percentage. The number for each analyzed plates is described in the figure legends as "n = x".

### RNAi screening for the ChR2(H134R)-induced avoidance assay

The *ce314 n744; uIs57; tmIs825* animals were examined, and the *ce314 n744; uIs57* and *ce314; tmIs825* animals were used as the negative controls. We selected 210 neural transcription factors that expressed in a neuron-selective manner from the WormBase (http://www.wormbase.org, WS220) and OrthoList [52]. Interference RNA feeding was performed as previously described [53]. The changes for the optogenetics assay were as follows: we plated 2 μl of 100 mM all-trans retinal (ATR) (Sigma-Aldrich, R2500) dissolved in 70 μl of OP50 culture medium to NGM plates containing 1 mM isopropyl-β-thiogalactoside and 100 μM ampicillin. After the plates were incubated for 12–16 h at 25°C in the dark, a L4 larva was placed on the plate and cultured for 4 days at 20°C in the dark. Then, 10–20 L4 and day-1 adult progenies per plate were manually and individually irradiated with 25% blue light using CFP and an ND filter (0.395 μw/cm^2^) at the heads for 2-sec. Manual scoring yielded data for an average of 10–20 worms per plate. Percent change was calculated by normalizing to the rates of avoidance behaviors of the animals during 2-sec stimulation that were treated with negative control RNAi (empty L4440 vector), because the RNAi-sensitive animals tended to have decreased transgene expression levels [54]. The experimenter was blinded to the nematode strains and RNAi-treated clones. We analyzed the types of avoidance behaviors of approximately 10 or 20 animals per experiment and calculated the frequency of each behavior.

### Osmotic ring assay

Ring assays were performed as previously described [55]. Briefly, 5–20 adult animals were placed on an agar plate within a 1.5-cm diameter ring containing 15 μl of 2 M sorbitol dissolved in water or water alone as a control. The osmotic avoidance index was defined as the fraction of animals that were inside the ring after 10 min. As the control experiment of rescue experiments (Figs 2D, 5C and 5D), we used *tmEx3905* as a control, which expressed the *Pinx-1*::*Venus* marker but not *Punc-130*::*LIN-32* (S4 Appendix). We performed the experiments on at least three different days and calculated the average percentage. The number for each analyzed plate is described in the figure legends as "n = x".

### Microscopy

Nematodes were immobilized with 50 mM sodium azide in M9 buffer on a 5% agarose pad containing 10 mM sodium azide. Confocal microscopic images were captured with Zeiss LSM710 confocal microscopes. Z-stacks spanning the focal depths (1 μm/step) of the neurons were collected with either a 40X water immersion or 63X oil immersion objective, and single-plane projections were generated with ZEN 2011 software (Zeiss, https://www.zeiss.co.jp/microscopy/downloads/zen.html). Additional fluorescence images and differential interference contrast images were obtained using a BX51 microscope equipped with a DP30BW CCD camera (Olympus Optical).

### Dye filling

We performed dye-filling assays as described in a previous report [56]. The assay was performed by incubating the day-1 adults in Dil solution (10 μg/ml 1,1'-Dioctadecyl-3,3,3',3'-Tetramethylindocarbocyanine Perchlorate (DiIC_18_(3) in M9 buffer) (Molecular Probes, Eugene, OR, USA V-22885) for 30 min at room temperature. Then, animals were cultured on NGM plates seeded with OP50 for 1 h and observed under a fluorescence microscope. The number for each analyzed individual is described in the figure legends as "n = x".

### Calcium imaging of neurons

Calcium imaging was performed using the olfactory chip method as previously described [57]. We used S basal as a buffer solution and 2 M sorbitol dissolved in S basal as an osmotic stimulant. Optical recordings of ASH and other neurons were performed on a BX51 microscope with a 60X Olympus oil immersion objective and a CoolSnap CCD camera (Photometrics) or an IX71 microscope with a 40X immersion objective (Olympus Optical) and an ORCA-Flash2.8 CMOS camera (Hamamatsu photonics), respectively, and analyzed with MetaMorph software (Molecular Devices). We captured time stacks of the fluorescence images at 1 frame per second. Images were analyzed as previously described [58]. We calculated the Percent change in the fluorescence intensity relative to the average intensity during the 5 sec before stimulation. Image tracking was performed using a custom ImageJ (NIH, https://imagej.nih.gov/ij/) plug-in. A rectangular region of interest (ROI) was drawn surrounding the cell body, and for every frame, the ROI was shifted according to the new position at the center.

### Histamine-HisCl assay

Histamine-HisCl assays were performed as previously described [16]. Assays were performed blind. We analyzed the types of avoidance behaviors of approximately 10 animals per experiment, and we calculated their frequency. We classified the types of avoidance behaviors of approximately 10 animals per plate and calculated the percentage of each behavior. We performed the experiments on at least three different days and calculated the average percentage. The number for each analyzed plate is described in the figure legends as "n = x".

### Calcium imaging of the neck muscles

We performed the drop test as described above under a dim light to identify pharynx locations, and images were collected with an ORCA-Flash2.8 CMOS camera and HCImage software (Hamamatsu photonics, https://hcimage.com/download/) at 45 frames per second. We analyzed the images from 1 second before the time of contact with the drop to the end of the backward behavior. Peak fluorescence was calculated from the following equation: ΔF/F_min_ = (F_max_—F_min_)/F_min_, where F_min_ and F_max_ were the actual fluorescence intensity values of a circular ROI in the center of the ventral neck muscles for every frame. Areas were defined as the induced fluorescence rate of the body length. The number for each analyzed individual (animals) is described in the figure legends as "n = x".

### Quantification and statistical analysis

#### Statistics

Statistical analyses were performed using GraphPad Prism7 software (GraphPad Software, Inc, http://www.graphpad.com/). Pairwise comparisons of frequencies of omega turn within only two groups or multiple groups were carried out via analysis of Fisher's exact test (Fig 4B, S5A, S5E and S5G Appendix, S3 Table), Student’s *t*-tests (Fig 3F and 3G, S5F Appendix) or variance (ANOVA) followed by the Tukey's post hoc tests (other than described above). All histogram data were obtained from three or more independent experiments and are presented as the mean ± SEM. In both cases, "n" is the number of plates (cohorts) of approximately 10–20 animals each. The error bars of scatter plot data presented as the mean ± SD. In Figs 4B and 6, S5A, S5E and S5G Appendix and S3 Table, "n" is the number of individuals (animals). Statistical information and the total number of experiments, animals, or cells analyzed per experiment are also provided in the figure legends.

## Supporting information

S1 Table A list of all screened transcription factors. A list of all screened transcription factors in the RNAi screening. Percent change was calculated by normalizing to the rates of avoidance behaviors of the animals during 2-sec stimulation that were treated with negative control RNAi (empty L4440 vector). Note that we did not distinguish the type of avoidance behavior in the screening.
(XLSX)

S2 Table Eighteen candidate transcription factors are involved in avoidance behavior. A list of candidate transcription factors in the screenings. We performed three screenings: a combination of optogenetics and enhanced neuronal RNAi methods ("RNAi & ChR2"); optogenetics or the high osmolarity ring assays using each mutant of the candidate genes ("Mutant & ChR2" and "Mutant & Osmolarity"). Percent change was calculated by normalizing to the rates of avoidance behaviors of the animals during 2-sec stimulation that were treated with negative control RNAi (empty L4440 vector), or that of wild type animals. N/A: not yet analyzed.
(TIF)

S3 Table Summary of cell differentiation defects of avoidance circuits in *lin-32* and *fax-1* mutants. The rates of normal cells and analyzed neuron numbers are shown. The deficiencies were defined as the following: a change in the number of marker-positive cells and/or reduced intensity. *lin-32* mutants showed differentiation defects in the AIB neurons, while the other neurons seemed to be normal. n means the number of individuals (animals). N/A: not yet analyzed. *p < 0.05, Fisher’s exact test.
(TIF)

S1 Appendix Results of the control experiments. (A) Avoidance behaviors of ASH::ChR2(H134R)*; lite-1* animals without food with the 2-sec stimulation. n = 7,7,6,10,9,9.
(B) The *lite-1* mutants as a negative control with the 2-sec stimulation. n = 4,7,7,4,7,7.
(C) Wild type animals adjust probability of omega turns depending on the glycerol drop concentration. n = 6 each.
(D) ASH::ChR2(H134R); *lin-32 lite-1* animals showed reduced omega turns even without food. n = 7 each.
(E) *lin-32* animals show reduced omega turns independent on the glycerol drop concentration. n = 6 each.
n = plate (cohort) of approximately 10–20 animals. The data are presented as the mean ± SEM.
(TIF)

S2 Appendix Normal differentiation of ASH sensory neurons, AIA, AVA and RIM interneurons, and DA/VA motor neurons in the *lin-32* and *fax-1* mutants. (A-C) The normal expression pattern of *Psra-6*::*GFP*, which is expressed in the ASH sensory neurons and faintly expressed in the ASI neurons of all animal types (arrowheads).
(D-F) Normal dye-filling patterns, which suggest that ASH in both animal types have functional cilia and dendrite for transport (arrowhead).
(G,H) Normal expression of *Peat-4*::*mCherry* marker (arrowhead) supports normal glutamatergic synaptic transmission of ASH. N/A, not analyzed.
(I-K) The normal expression pattern of AIA marker *Pgcy-28(d)*::*EGFP* (arrowhead).
(L-N) The expression pattern of *Pnmr-1*::*GFP* and *Pglr-1*::*DsRed2*. Double-labeled neurons (arrowheads) include AVA, AVE, AVD, and RIM (arrowhead).
(O-Q) The normal expression pattern of DA/VA motor neuronal marker, *Pdel-1*::*GFP*.
Scale bars, 10 μm.
(TIF)

S3 Appendix Cells in the AIB sister lineage normally differentiate in the *lin-32* mutants. (A) Schematic of the ABpl/raapap cell lineage. AIB shares a common precursor cell with AWA, ASG and ASI sensory neurons. The sister cells of ASI are removed via programmed cell death.
(B) Schematic of the wild type AIB, AWA, ASG, and ASI morphologies (green).
(C-H) ASI, AWA, and ASG show normal morphologies, numbers, and marker expression levels in wild type animals and *lin-32* mutants.
Asterisks show the cells on the opposite side. Scale bars, 10 μm.
(TIF)

S4 Appendix AIB marker-positive cells in the *lin-32* mutants show functional defects. *lin-32* animals expressing AIB marker *Pinx-1*::*Venus* ("*lin-32* AIB marker +") show defects that are comparable to those of the AIB marker-negative siblings ("*lin-32* AIB marker -"). *p < 0.05, ANOVA followed by the Tukey's post hoc tests. n = 6,3,8,4.
n = plate (cohort) of approximately 10–20 animals. The data are presented as the mean ± SEM.
(TIF)

S5 Appendix AIB excitation correlates omega turn via gap junction. (A) *lite-1* mutants expressing ChR2(H134R) in AIB neurons (AIB::ChR2(H134R); *lite-1*) with ATR exhibit omega turns even if without food condition. n = 50 each. n means the number of individuals (animals).
(B) Behavioral frequencies during 2- or 5-sec 100% light stimulations in ASH::ChR2-expressing animals. All animals exhibited avoidance behaviors. Almost all animals continued backward behaviors during stimulation. n = 8 (2 secs), n = 9 (5 secs). n = plate (cohort) of approximately 10–20 animals. The data are presented as the mean ± SEM.
(C) Timing of omega turns during the introduction or removal of the 100% light stimulus in ASH::ChR2-expressing animals. n = 6 (2 secs), n = 7 (5 secs). n = plate (cohort) of approximately 10–20 animals. The data are presented as the mean ± SEM.
(D) AIB::INX-1 transgenic animals as a control showed a similar result to wild type animals (Fig 1D). n = 6 each. n = plate (cohort) of approximately 10–20 animals. The data are presented as the mean ± SEM.
(E) AIB::mCherry transgenic animals as a control showed a similar result to wild type animals. n = 40, 59. n means the number of individuals (animals).
(F) Mean number of reversals in 3 min using free-moving wild type animals and AIB::TeTx transgenic animals. ***p < 0.001, Student's *t*-tests. n = 59, 60. n means the number of individuals (animals).
(G) AIB::UNC-1(n494) transgenic animals show similar avoidance behaviors to 2 M sorbitol. n = 60, 60. n means the number of individuals (animals).
(TIF)

S1 Movie Avoidance behaviors of the ASH::ChR2(H134R); lite-1 animals. Avoidance behaviors of the ASH::ChR2(H134R); *lite-1* animals are shown. They exhibited omega turns under 100% light irradiation and short reversals under 25% irradiation.
(MOV)

S2 Movie Avoidance behaviors of the ASH::ChR2(H134R); *lin-32* lite-1 animals. Avoidance behaviors of the ASH::ChR2(H134R); *lin-32 lite-1* animals are shown. They exhibited reversals under 100% light irradiation and no avoidance under 25% light irradiation.
(MOV)

S3 Movie Calcium concentration changes in the muscles of *Pmyo-3*::*G-CaMP2* transgenic animals. Calcium concentration changes in the muscles of *Pmyo-3*::*G-CaMP2* transgenic animals during the three types of avoidance behaviors in the drop tests.
(MOV)

S4 Movie Calcium concentration changes in the muscles of *lin-32; Pmyo-3*::*G-CaMP2* transgenic animals. Calcium concentration changes in the muscles of *lin-32*; *Pmyo-3*::*G-CaMP2* transgenic animals during the three types of avoidance behaviors in the drop tests.
(MOV)

S1 Dataset S1_Dataset file contains the all relevant datasets which were analyzed for graphs or summary statistics in the research described in this paper. (XLSX)
