# Supplementary figures and images for: OFF-responses of interneurons optimize avoidance behaviors depending on stimulus strength via electrical synapses
Source: PLoS Genet. 2018 Jun 25;14(6):e1007477. doi: 10.1371/journal.pgen.1007477 (PMC6034901; doi:10.1371/journal.pgen.1007477)

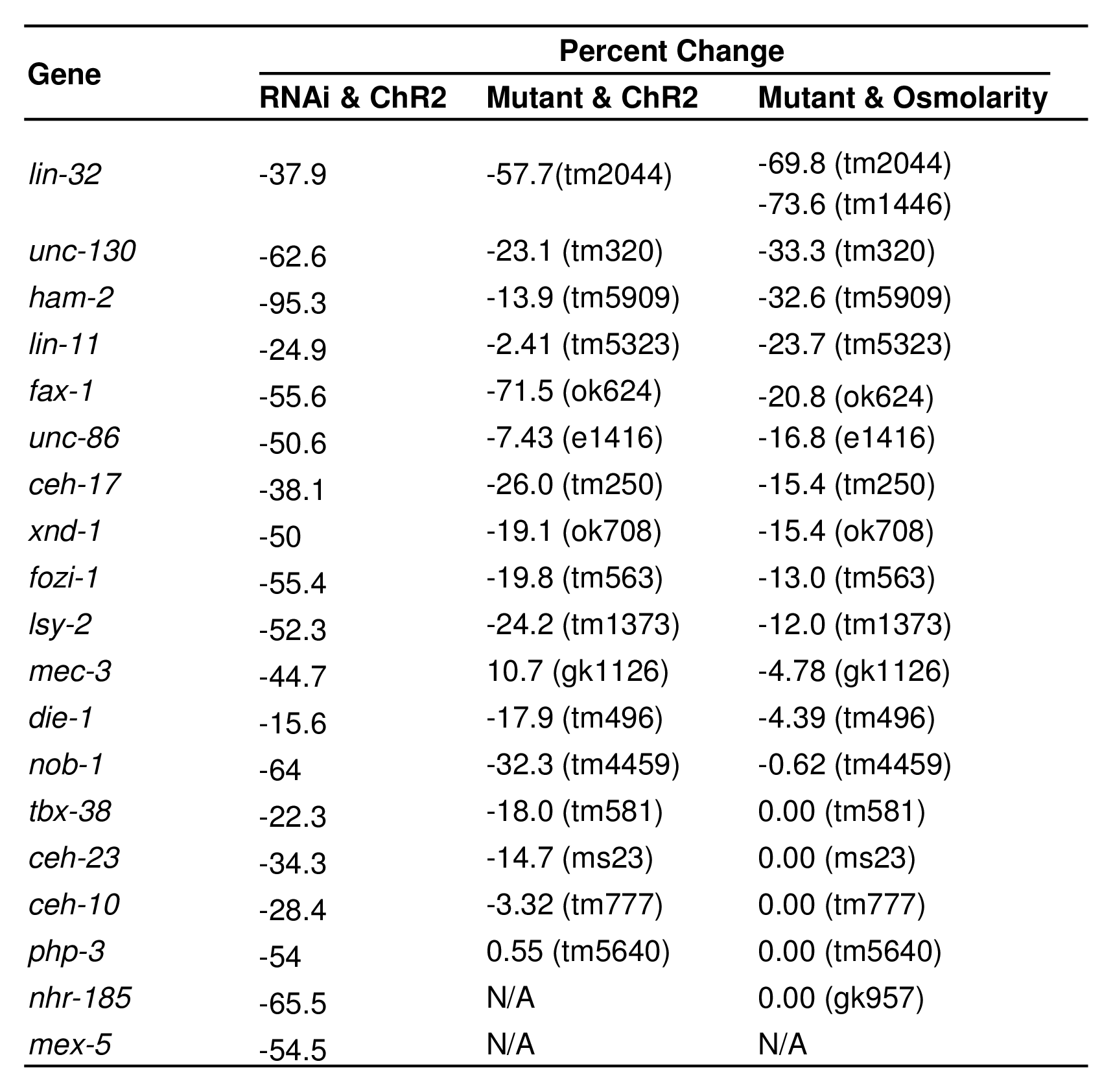

Supplement: S2 Table — A list of candidate transcription factors in the screenings. We performed three screenings: a combination of optogenetics and enhanced neuronal RNAi methods ("RNAi & ChR2"); optogenetics or the high osmolarity ring assays using each mutant of the candidate genes ("Mutant & ChR2" and "Mutant & Osmolarity"). Percent change was calculated by normalizing to the rates of avoidance behaviors of the animals during 2-sec stimulation that were treated with negative control RNAi (empty L4440 vector), or that of wild type animals. N/A: not yet analyzed. (TIF) [file pgen.1007477.s002.tif]

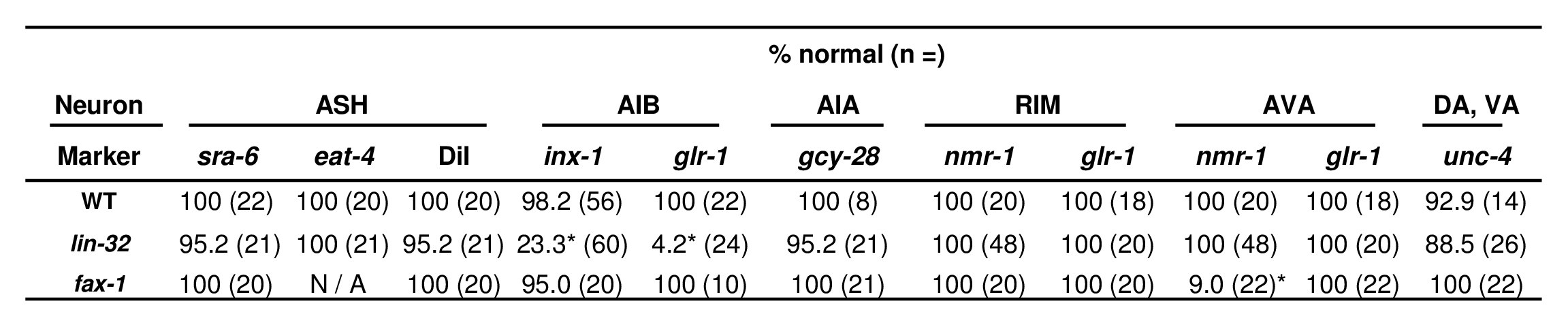

Supplement: S3 Table — The rates of normal cells and analyzed neuron numbers are shown. The deficiencies were defined as the following: a change in the number of marker-positive cells and/or reduced intensity. lin-32 mutants showed differentiation defects in the AIB neurons, while the other neurons seemed to be normal. n means the number of individuals (animals). N/A: not yet analyzed. *p < 0.05, Fisher’s exact test. (TIF) [file pgen.1007477.s003.tif]

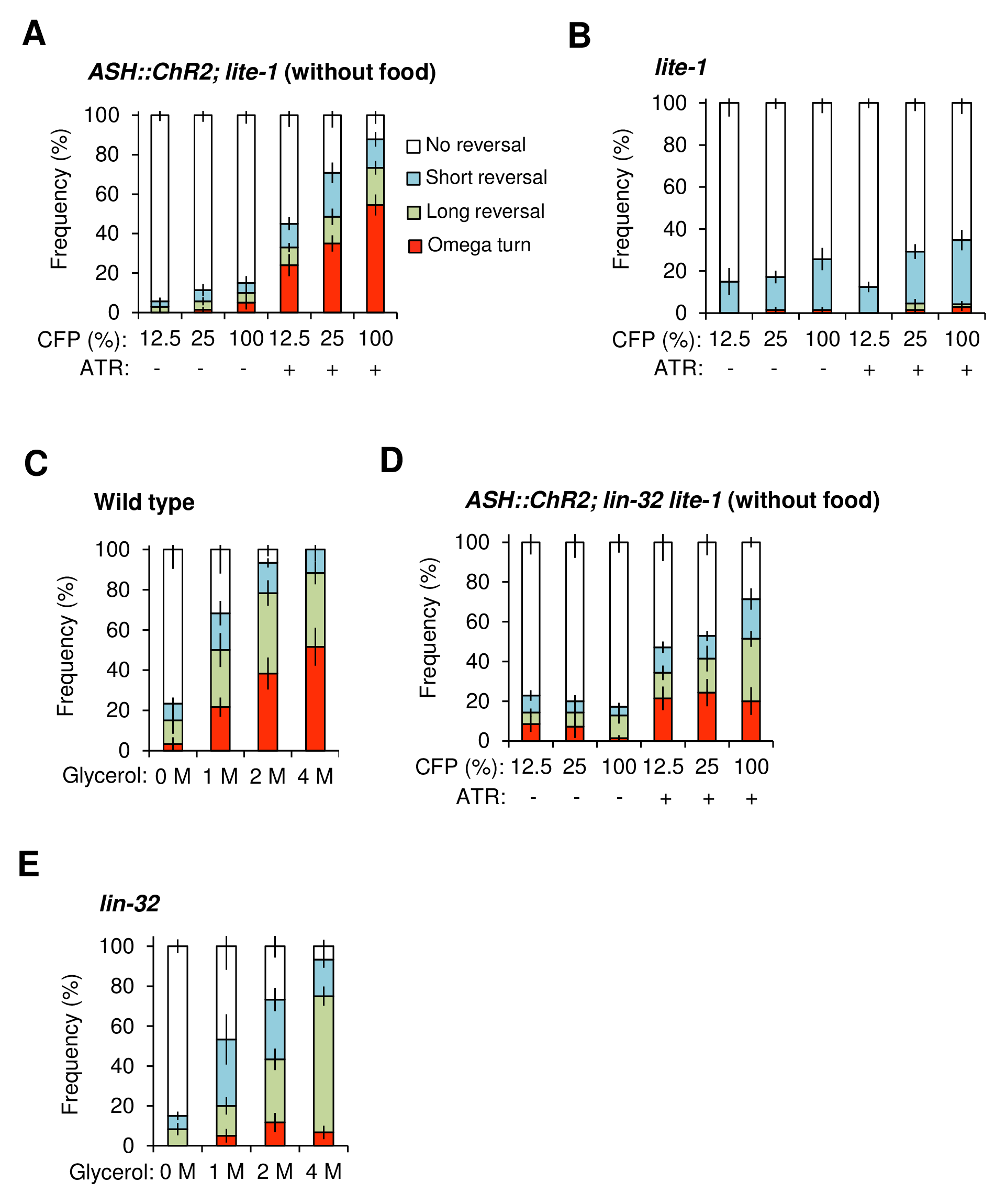

Supplement: S1 Appendix — (A) Avoidance behaviors of ASH::ChR2(H134R); lite-1 animals without food with the 2-sec stimulation. n = 7,7,6,10,9,9. (B) The lite-1 mutants as a negative control with the 2-sec stimulation. n = 4,7,7,4,7,7. (C) Wild type animals adjust probability of omega turns depending on the glycerol drop concentration. n = 6 each. (D) ASH::ChR2(H134R); lin-32 lite-1 animals showed reduced omega turns even without food. n = 7 each. (E) lin-32 animals show reduced omega turns independent on the glycerol drop concentration. n = 6 each. n = plate (cohort) of approximately 10–20 animals. The data are presented as the mean ± SEM. (TIF) [file pgen.1007477.s004.tif]

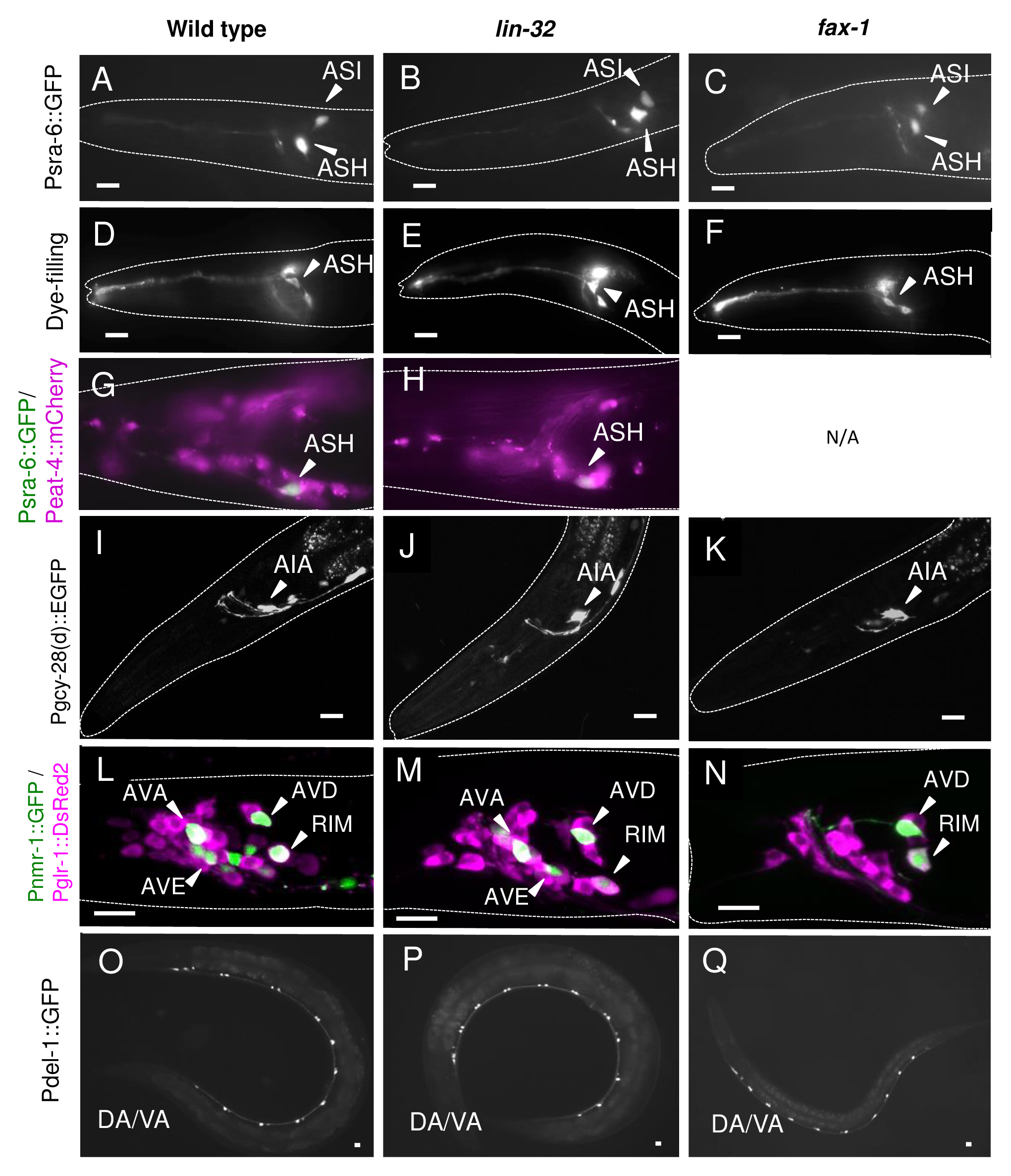

Supplement: S2 Appendix — (A-C) The normal expression pattern of Psra-6::GFP, which is expressed in the ASH sensory neurons and faintly expressed in the ASI neurons of all animal types (arrowheads). (D-F) Normal dye-filling patterns, which suggest that ASH in both animal types have functional cilia and dendrite for transport (arrowhead). (G,H) Normal expression of Peat-4::mCherry marker (arrowhead) supports normal glutamatergic synaptic transmission of ASH. N/A, not analyzed. (I-K) The normal expression pattern of AIA marker Pgcy-28(d)::EGFP (arrowhead). (L-N) The expression pattern of Pnmr-1::GFP and Pglr-1::DsRed2. Double-labeled neurons (arrowheads) include AVA, AVE, AVD, and RIM (arrowhead). (O-Q) The normal expression pattern of DA/VA motor neuronal marker, Pdel-1::GFP. Scale bars, 10 μm. (TIF) [file pgen.1007477.s005.tif]

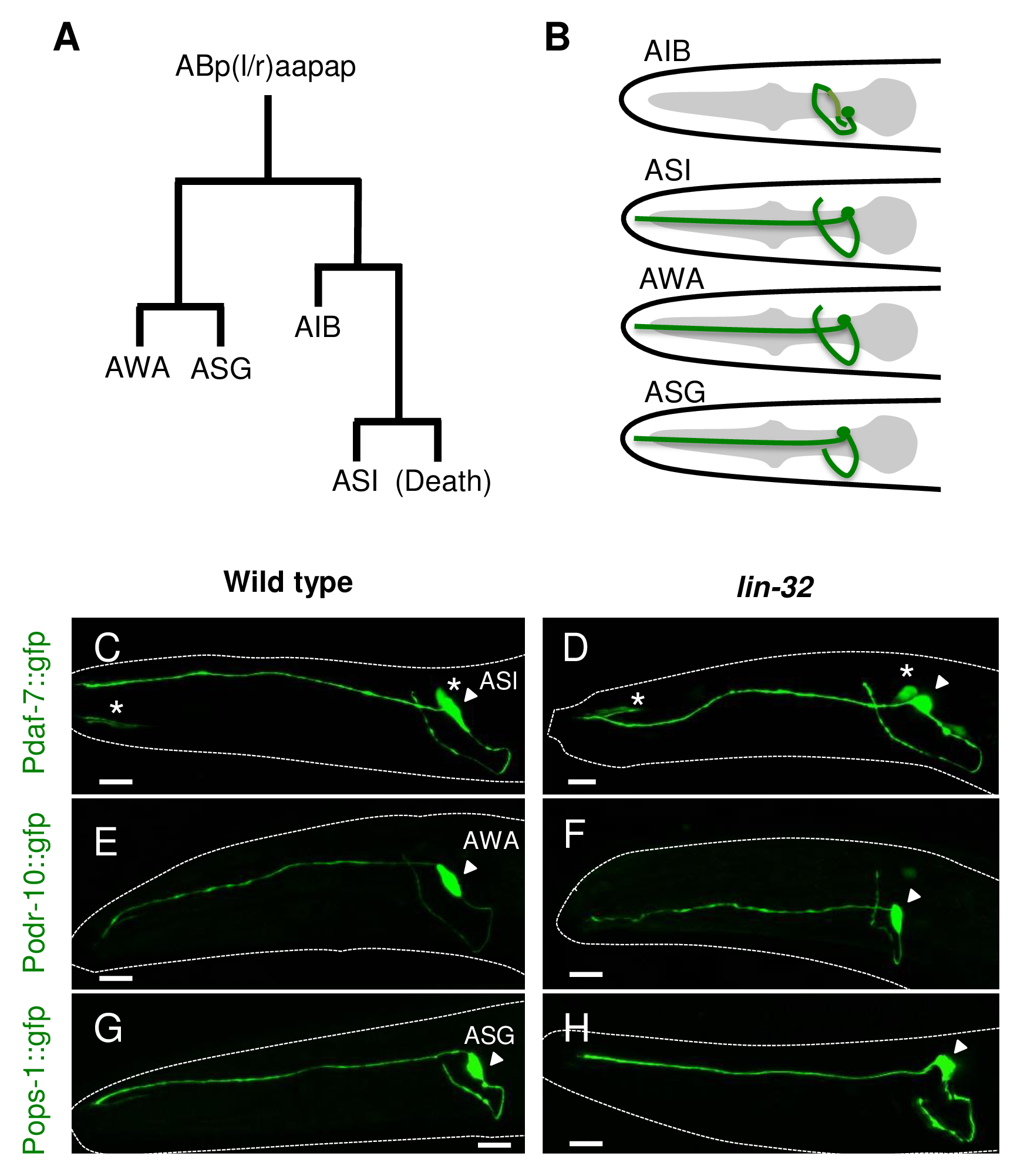

Supplement: S3 Appendix — (A) Schematic of the ABpl/raapap cell lineage. AIB shares a common precursor cell with AWA, ASG and ASI sensory neurons. The sister cells of ASI are removed via programmed cell death. (B) Schematic of the wild type AIB, AWA, ASG, and ASI morphologies (green). (C-H) ASI, AWA, and ASG show normal morphologies, numbers, and marker expression levels in wild type animals and lin-32 mutants. Asterisks show the cells on the opposite side. Scale bars, 10 μm. (TIF) [file pgen.1007477.s006.tif]

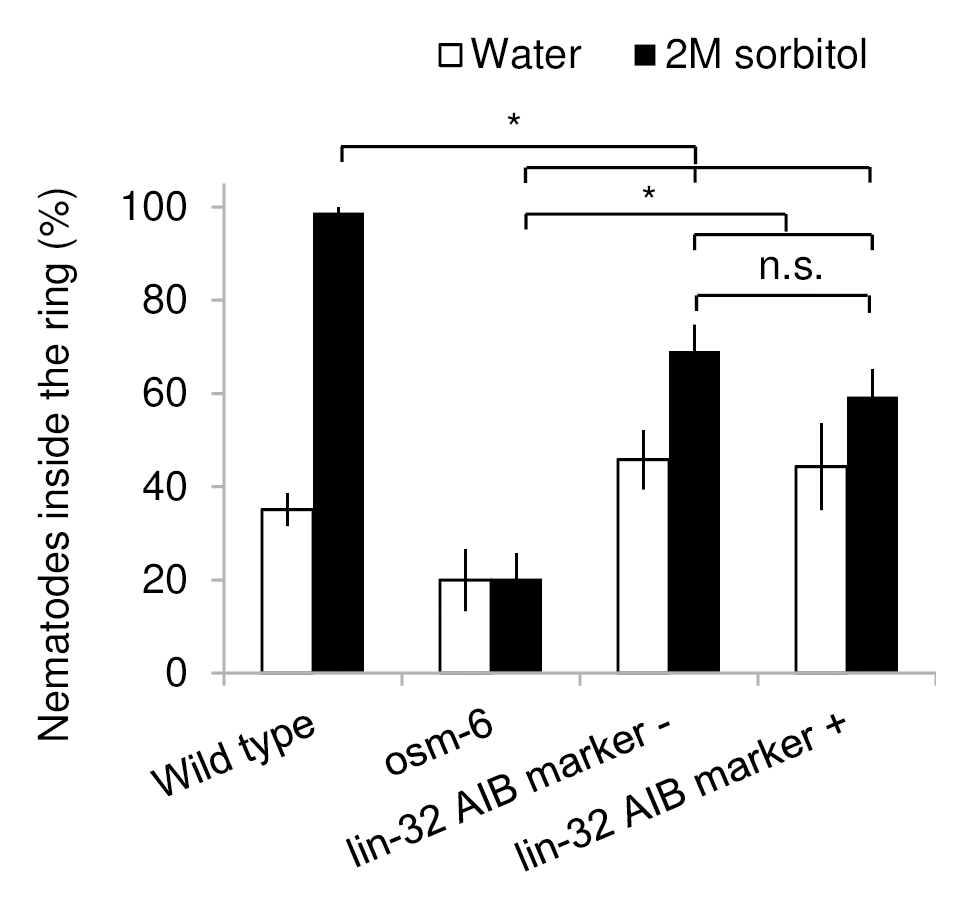

Supplement: S4 Appendix — lin-32 animals expressing AIB marker Pinx-1::Venus ("lin-32 AIB marker +") show defects that are comparable to those of the AIB marker-negative siblings ("lin-32 AIB marker -"). *p < 0.05, ANOVA followed by the Tukey's post hoc tests. n = 6,3,8,4. n = plate (cohort) of approximately 10–20 animals. The data are presented as the mean ± SEM. (TIF) [file pgen.1007477.s007.tif]

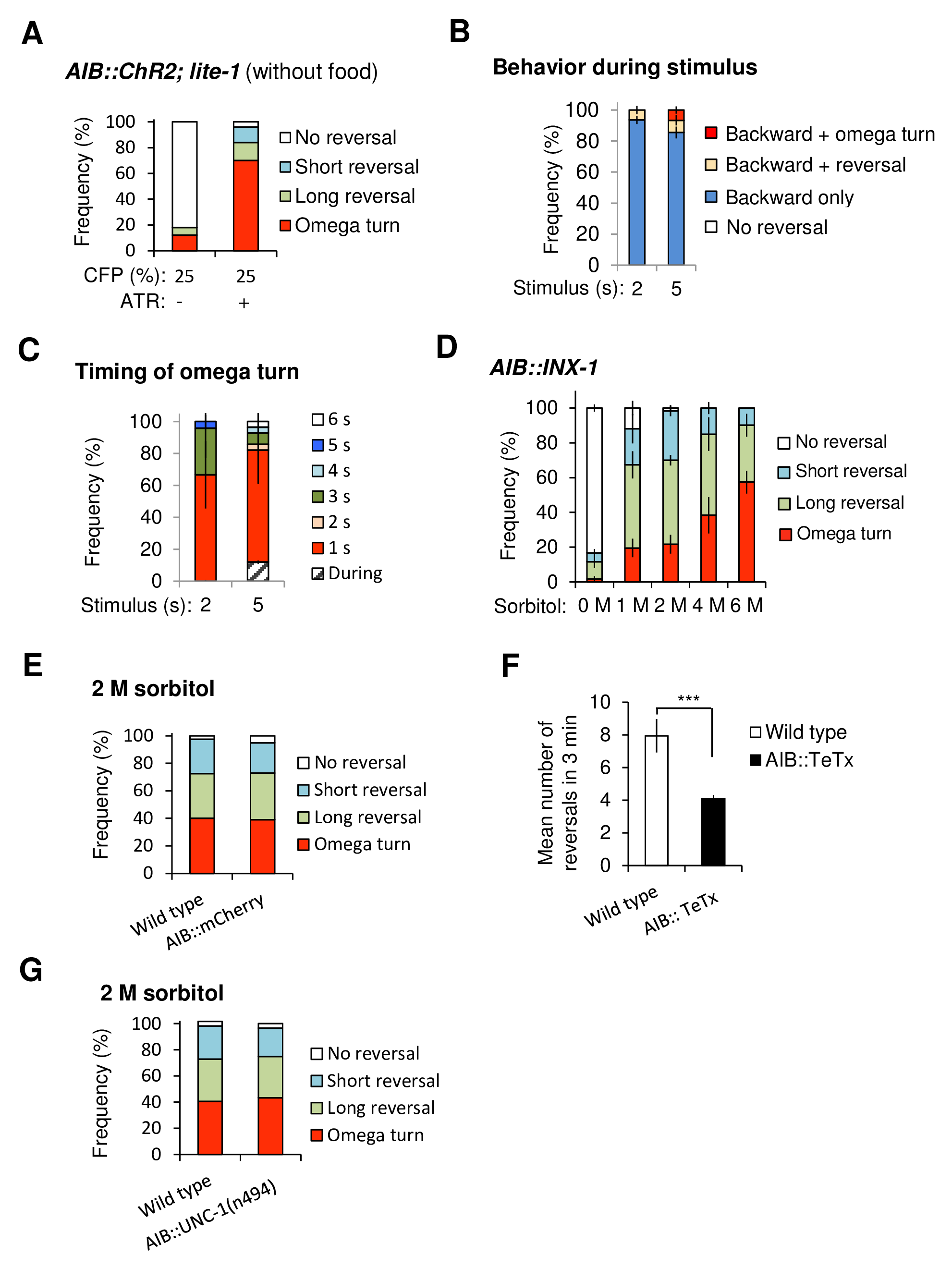

Supplement: S5 Appendix — (A) lite-1 mutants expressing ChR2(H134R) in AIB neurons (AIB::ChR2(H134R); lite-1) with ATR exhibit omega turns even if without food condition. n = 50 each. n means the number of individuals (animals). (B) Behavioral frequencies during 2- or 5-sec 100% light stimulations in ASH::ChR2-expressing animals. All animals exhibited avoidance behaviors. Almost all animals continued backward behaviors during stimulation. n = 8 (2 secs), n = 9 (5 secs). n = plate (cohort) of approximately 10–20 animals. The data are presented as the mean ± SEM. (C) Timing of omega turns during the introduction or removal of the 100% light stimulus in ASH::ChR2-expressing animals. n = 6 (2 secs), n = 7 (5 secs). n = plate (cohort) of approximately 10–20 animals. The data are presented as the mean ± SEM. (D) AIB::INX-1 transgenic animals as a control showed a similar result to wild type animals (Fig 1D). n = 6 each. n = plate (cohort) of approximately 10–20 animals. The data are presented as the mean ± SEM. (E) AIB::mCherry transgenic animals as a control showed a similar result to wild type animals. n = 40, 59. n means the number of individuals (animals). (F) Mean number of reversals in 3 min using free-moving wild type animals and AIB::TeTx transgenic animals. ***p < 0.001, Student's t-tests. n = 59, 60. n means the number of individuals (animals). (G) AIB::UNC-1(n494) transgenic animals show similar avoidance behaviors to 2 M sorbitol. n = 60, 60. n means the number of individuals (animals). (TIF) [file pgen.1007477.s008.tif]
